# Development and Characterization of Innovative Multidrug Nanoformulation for Cardiac Therapy

**DOI:** 10.3390/ma16051812

**Published:** 2023-02-22

**Authors:** Amandine Gendron, Séverine Domenichini, Sandrine Zanna, Frédéric Gobeaux, Christophe Piesse, Didier Desmaële, Mariana Varna

**Affiliations:** 1 Université Paris-Saclay, CNRS UMR 8612, Institut Galien Paris-Saclay, 91400 Orsay, France; 2 UMS-IPSIT Plateforme MIPSIT, Université Paris-Saclay, CNRS, Inserm, Ingénierie et Plateformes au Service de l’Innovation Thérapeutique, 91400 Orsay, France; 3 PSL Research University, Chimie ParisTech-CNRS, Institut de Recherche de Chimie Paris, Research Group Physical Chemistry of Surfaces, 11 rue Pierre et Marie Curie, 75005 Paris, France; 4 Université Paris-Saclay, CEA, CNRS, NIMBE, 91191 Gif-sur-Yvette, France; 5 Sorbonne Université, CNRS, Institut de Biologie Paris-Seine (IBPS), Plateforme d’Ingénierie des Protéines—Service de Synthèse Peptidique, 75005 Paris, France

**Keywords:** bioconjugate, therapeutic peptide, nanoparticles, cytotoxicity, antioxidant capacity

## Abstract

For several decades, various peptides have been under investigation to prevent ischemia/reperfusion (I/R) injury, including cyclosporin A (CsA) and Elamipretide. Therapeutic peptides are currently gaining momentum as they have many advantages over small molecules, such as better selectivity and lower toxicity. However, their rapid degradation in the bloodstream is a major drawback that limits their clinical use, due to their low concentration at the site of action. To overcome these limitations, we have developed new bioconjugates of Elamipretide by covalent coupling with polyisoprenoid lipids, such as squalenic acid or solanesol, embedding self-assembling ability. The resulting bioconjugates were co-nanoprecipitated with CsA squalene bioconjugate to form Elamipretide decorated nanoparticles (NPs). The subsequent composite NPs were characterized with respect to mean diameter, zeta potential, and surface composition by Dynamic Light Scattering (DLS), Cryogenic Transmission Electron Microscopy (CryoTEM) and X-ray Photoelectron Spectrometry (XPS). Further, these multidrug NPs were found to have less than 20% cytotoxicity on two cardiac cell lines even at high concentrations, while maintaining an antioxidant capacity. These multidrug NPs could be considered for further investigations as an approach to target two important pathways involved in the development of cardiac I/R lesions.

## 1. Introduction

The reduction or cessation of blood flow in the coronary arteries results in a reduced supply of nutrients and oxygen to the heart muscle tissue, causing cardiac ischaemia. Over time, this ischaemia will lead to cell death and damage to the heart muscle, known as acute myocardial infarction (AMI). AMI is one of the leading causes of death worldwide [1], with approximately 16 million deaths per year [2]. Fortunately, thrombolytic therapy or primary percutaneous coronary intervention (PCI) can be used to reperfuse the heart tissue, reducing the size of the infarct. However, reperfusion itself may cause additional cellular and tissue damage, known as ischemia/reperfusion (I/R) injury, which could be prevented by adequate cardioprotection [3]. One of the main effectors of I/R injury is a pore located in the mitochondria called mitochondrial permeability transition pore (mPTP). In recent decades, various therapeutic peptides have been evaluated as possible cardioprotective agents, including elamipretide and cyclosporin A.

Since its discovery in 2004 [4], elamipretide (SS-31, MTP-131 or Bendavia) [2] has attracted the attention of the scientific and medical community. Due to its small size, ease of synthesis, and water solubility, elamipretide is a potential therapeutic peptide [5]. Its structural motif is based on alternating aromatic and basic amino acid residues [4], allowing cellular permeability and targeted delivery to the inner mitochondrial membrane (IMM) [4,5]. This interest is also related to the fact that this peptide has hydrogen peroxide and peroxynitrite scavenging properties [2,5], thus reducing intracellular reactive oxygen species (ROS) [4,5] and inhibiting lipid peroxidation [2,5,6].

In vitro studies have shown that this peptide can be taken up by cells, even with a net charge of +3 at physiological pH [7] and exhibits tropism for cardiolipin by concentrating up to 5000-fold in the IMM [4,7]. Moreover, it has the ability to decrease mitochondrial ROS production, preventing the opening of the mitochondrial permeability transition pore (mPTP), and thus the release of the cytochrome c [4]. Elamipretide has therefore been tested in different ischaemia/reperfusion (IR) models. Kloner et al. showed a reduced infract size in an ex vivo guinea pig IR model and an in vivo sheep IR model [2,8]. Recently, Allen et al. demonstrated improved mitochondrial function in an ex vivo rat IR model [9].

Despite these encouraging results, the clinical data from phase 2a trial EMBRACE STEMI remained mitigated as the drug administered to patients just prior reperfusion (i.e., PCI) was well tolerated but was not associated with a reduction in infarct size [10]. This could be explained by the quick degradation of the peptide in the bloodstream, although some modifications have been made to reduce the degradation of this peptide, such as a switch from an L-amino acid to a D-amino acid in the first position to make it resistant to aminopeptidase activity and a C-terminal amidation to reduce hydrolysis [5,11].

To overcome the poor pharmacokinetic of the free peptide SS-31, some teams have proposed to encapsulate them in nanoparticles using different types of biodegradable and biocompatible polymers via covalent [12,13] or ionic bond [14,15]. Indeed, the potential of nanotechnologies has increased tremendously in recent years, leading to very innovative applications in the medical field, not only for drug delivery and theragnostic applications [16,17,18], but also for targeted delivery of pharmaceutics using microrobots [19].

**Figure 1:**
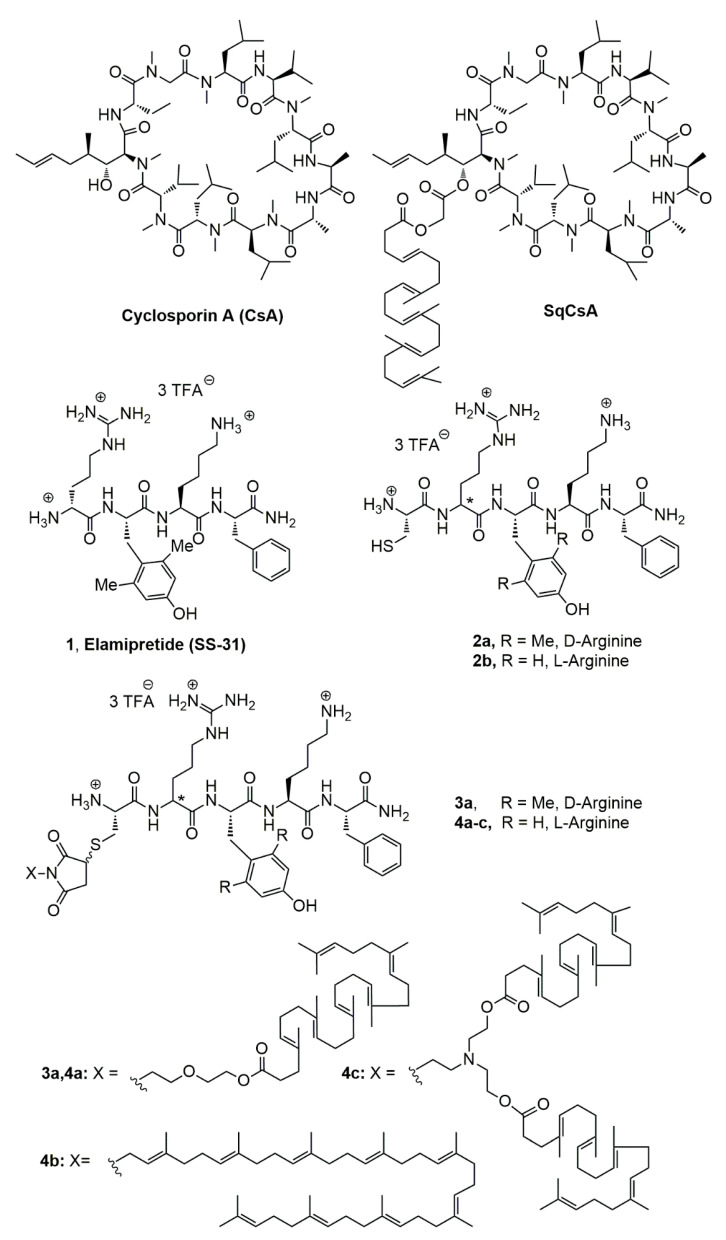
Structures of cyclosporin A (CsA), cyclosporin squalene conjugate (SqCsA), Elamipretide (SS-31), the cysteine bound peptides **2a**,**b** and the poylyisoprenyl conjugates **3a**–**c**, **4a**. The chiral carbon atom of arginine is marked with an asterisk *.

We report herein multidrug nanoparticles based on bioconjugation of CsA and elamipretide with polyisoprenoid derivatives (i.e., squalene or solanesol) (Figure 1). These multidrug nanoparticles were fully characterized with respect to size, zeta potential, stability, and surface composition by means of various physico-chemical methods. We further demonstrated that they preserved the antioxidant capacity of the elamipretide without cytotoxicity on the cardiac cell lines tested.

## 2. Materials and Methods

### 2.1. Materials

Furan, maleic anhydride, 1-ethyl-3-(3-dimethylaminopropyl)carbodiimide (EDCI), 2-(2-aminoethoxy)ethan-1-ol, hydrochloric acid, the H9c2 cell line, Dulbecco’s Modified Eagle’s Medium (DMEM) D5796 and D6429 high glucose, Dulbecco’s Phosphate-Buffered Saline (PBS), 4-(2-hydroxyethyl)-1-piperazineethanesulfonic acid (HEPES; 1 M, pH 7.0–7.6), penicillin–streptomycin, D-(+)-glucose, thiazolyl blue tetrazolium bromide (MTT) were purchased from Sigma-Aldrich (Lyon, France). Fetal Bovine Serum (FBS) was purchased from Life Technologies (Illkirch-Graffenstaden, France). The MCEC cell line was obtained from Tebu-Bio (Le Perray en Yvelines, France). ORAC assay kit was obtained from Abcam (Cambridge, MA, USA). 4-(Dimethylamino)pyridine (DMAP) and 2,2′-[(2-aminoethyl)imino]diethanol were purchased from Alfa Aesar (Kandel, Germany). Diisopropyl azodicarboxylate (DIAD) was obtained from Acros Organics (Illkirch, France). Cyclosporin A squalene conjugate (SqCsA) was synthetized as previously described [20]. Peptides Cys-Arg-Tyr-Lys-Phe-NH_2_ (**2b**) and Cys-D-Arg-2,6-diMe-Tyr-Lys-Phe-NH_2_ (**2a**) were kindly supplied by M. Piesse of the protein engineering facility of IBPS, Sorbonne University. Squalene acetic acid was prepared from commercially available squalene as previously reported [21]. cis-exo-7-Oxabicyclo [2.2.1]-5-heptene-2,3-dicarboxylic anhydride (**5**) was obtained from furan and maleic anhydride according to Woodward and Baer [22].

### 2.2. NMR Data of the Peptides

#### 2.2.1. NMR Data of [Cys-Arg-Tyr-Lys-Phe-NH2]^3+^ 3CF_3_CO_2−_ (**2b**, Figure 1)

^1^H NMR (400 MHz, CD_3_OD) *δ*: 7.29–7.23 (m, 4H, H-2′_Phe_, H-3′_Phe_, H-5′_Phe_, H-6′_Phe_), 7.21–7.15 (m, 1H, H-4′_Phe_), 7.06 (d, *J* = 8.4 Hz, 2H, H-2′_Tyr_, H-6′_Tyr_), 6.70 (d, *J* = 8.4 Hz, 2H, H-3′_Tyr_, H-5′_Tyr_), 4.59 (dd, *J* = 8.8 Hz, *J* = 6.0 Hz, 1H, H_αPhe_), 4.54 (dd, *J* = 9.0 Hz, *J* = 5.2 Hz, 1H, H_αTyr_), 4.33 (dd, *J* = 8.0 Hz, *J* = 6.0 Hz, 1H, H_αArg_), 4.23 (dd, *J* = 8.0 Hz, *J* = 6.4 Hz, 1H, H_αLys_), 4.08 (t, *J* = 5.8 Hz, 1H, H_αCys_), 3.20–3.11 (m, 3H, H_βPhe_, H_δArg_), 3.04–2.92 (m, 4H, H_βTyr_, H_βPhe_, H_βCys_), 2.88 (t, *J* = 7.6 Hz, 2H, H_εLys_), 2.82 (dd, *J* = 14.0 Hz, 9.0 Hz, 1H, H_βTyr_), 1.83–1.50 (m, 8H, H_βArg_, H_βLys_, H_γArg_, H_δLys_), 1.36–1.25 (m, 2H, H_γLys_); ^13^C NMR (100 MHz, CD_3_OD) *δ*: 175.8 (C, *C*ON_Phe_), 173.7 (C, *C*ON_Tyr_), 173.3 (2C, *C*ON_Arg_, *C*ON_Lys_), 168.62 (C, *C*ON_Cys_), 158.7 (C, N*C*=N_Arg_), 157.3 (C, C-4′_Tyr_), 138.3 (C, C-1′_Phe_), 131.4 (2CH, C-2′_Tyr_, C-6′_Tyr_), 130.5 (2CH, C-3′_Phe_, C-5′_Phe_), 129.5 (2CH, C-2′_Phe_, C-6′_Phe_), 128.72 (C, C-1′_Tyr_), 127.8 (CH, C-4′_Phe_), 116.4 (2CH, C-3′_Tyr_, C-5′_Tyr_), 56.3 (CH, CH_αTyr_), 55.8 (CH, CH_αPhe_ or CH_αCys_), 55.7 (CH, CH_αPhe_ or CH_αCys_), 54.8 (CH, CH_αArg_ or CH_αLys_), 54.7 (CH, CH_αArg_ or CH_αLys_), 41.9 (CH_2_, CH_2δArg_), 40.5 (CH_2_, CH_2εLys_), 38.8 (CH_2_, CH_2βPhe_), 37.9 (CH_2_, CH_2βTyr_), 32.3 (CH_2_, CH_2βLys_), 30.0 (CH_2_, CH_2βArg_), 28.0 (CH_2_, CH_2 δLys_), 26.4 (CH_2_, CH_2βCys_), 26.1 (CH_2_, CH_2γArg_), 23.5 (CH_2_, CH_2γLys_).

#### 2.2.2. NMR Data of [Cys-D-Arg-diMeTyr-Lys-Phe-NH2]^3+^ 3CF_3_CO_2−_ (**2a**, Figure 1)

^1^H NMR (400 MHz, CD_3_OD) *δ*: 7.29–7.23 (m, 5H, H _Phe_)_,_ 6.44 (s, 2H, H-3′_Tyr_, H-5′_Tyr_), 4.67 (dd, *J* = 9.6 Hz, *J* = 6.4 Hz, 1H, H _αMeTyr_), 4.53 (dd, *J* = 8.4 Hz, *J* = 6.4 Hz, 1H, H _αPhe_), 4.32 (t, *J* = 7.6 Hz, 1H, H _αArg_), 4.26 (t, *J* = 7.0 Hz, 1H, H _αLys_), 4.03 (t, *J* = 6.8 Hz, 1H, H_αCys_), 3.22–3.05 (m, 4H, H _βPhe_, H_βTyr_, H_δArg_), 3.04–2.86 (m, 4H, H _βPhe_, H_βTyr_, H_βCys_), 2.91 (t, *J* = 7.4 Hz, 2H, H_εLys),_ 2.27 (s, 6H, H _MeTyr_), 1.85–1.20 (m, 10H, H _βArg_, H _βLys_, H _γArg_, H _δLys_, H _γLys_); ^13^C NMR (100 MHz, CD_3_OD) *δ*: 175.8 (C, *C*ON_phe_), 173.4–173.2 (3C, *C*ON_MeTyr_, *C*ON_Arg_, *C*ON_Lys_), 168.9 (C, *C*ON_Cys_), 158.2 (C, N*C*=N_Arg_), 156.4 (C, C-4′_MeTyr_), 139.7 (C, C-1′_Phe_), 138.3 (C, C-1′_MeTyr_), 130.4 (2CH, C-3′_Phe_, C-5′_Phe_), 129.5 (2CH, C-2′_Phe_, C-6′_Phe_), 127.8 (CH, C-4′_Phe_), 126.2 (C, C-2′_MeTyr_, C-6′_MeTyr_), 116.2 (2CH, C-3′_MeTyr_, C-5′_MeTyr_), 56.1–56.0 (2CH, CH_αTyr_ and CH_αCys_), C55.1 (CH, CH _αLys_), 54.5 (CH, CH_αArg_), 54.3 (CH, CH_αPhe_), 42.4 (CH_2_, CH_2δArg_), 40.1 (CH_2_, CH_2εLys_), 38.2 (CH_2_, CH_2βPhe_), 37.9 (CH_2_, CH_2βTyr_), 32.4 (CH_2_, CH_2βArg_), 31.4 (CH_2_, CH_2βMeTyr_), 29.4 (CH_2_, CH_2βLys_), 27.4 (CH_2_, CH_2δLys_), 26.1–25.9 (2CH_2_, CH_2γArg_, CH_2βCys_), 23.5 (CH_2_, CH_2γLys_), 20.7 (2CH_3_, CH_3 MeTyr_).

### 2.3. Synthesis of the Lipid Peptide Bioconjugates

#### 2.3.1. General

IR spectra were obtained as a neat liquid or a solid on a Perkin Elmer Spectrum 2 FTIR or an IR-Affinity-1S Shimadzu spectrometer. Only significant absorptions were listed. The ^1^H and ^13^C NMR spectra were recorded on Bruker Avance 300 (Bruker, Les Ulis, France) (300 MHz and 75 MHz for ^1^H and ^13^C, respectively,) or Bruker Avance 400 (Bruker, Les Ulis, France) (400 MHz and 100 MHz for ^1^H and ^13^C, respectively,) spectrometers. The ^19^F spectra were recorded on Bruker AC 200 P (Bruker, Les Ulis, France) (188 MHz). Recognition of methyl, methylene, methine, and quaternary carbon nuclei in ^13^C NMR spectra rests on the *J*-modulated spin-echo sequence. Low resolution mass spectra were recorded on an Electrospray Ionization (ESI) LTQ-Orbitrap Velos Pro (ThermoFisher, San Jose, CA, USA). Thin layer chromatographies (TLCs) were performed using Merck silica gel 60 F254 glass precoated plates (0.25 mm layer). Column chromatography was performed on Merck silica gel 60 (230–400 mesh American Society for Testing and Materials (ASTM)). Toluene and Dichloromethane (DCM) were distilled from calcium hybrid under nitrogen atmosphere. All reactions involving air- or water-sensitive compounds were routinely conducted in glassware that was flame-dried under a positive pressure of nitrogen.

#### 2.3.2. Synthesis of 4-[2-(2-Hydroxyethoxy)ethyl]-10-oxa-4-azatricyclo [5.2.1.0^2,6^]dec-8-ene-3,5-dione (**6**) (Figure 2)

To a stirred solution of 4,10-dioxatricyclo [5.2.1.0^2,6^]dec-8-ene-3,5-dione (**5**) (2.86 g, 15.4 mmol) in ethanol (8 mL) at 0 °C was added dropwise a solution of triethylamine (1.71 g; 17.0 mmol) and 2-(2-aminoethoxy)ethanol (2.26 g; 22.4 mmol) in ethanol (5 mL). The reaction mixture was stirred at 0 °C for 1h and warmed up to 80 °C and stirred for a further 8 h. After cooling, the mixture was concentrated under vacuum, taken into CH_2_Cl_2_ (100 mL) and washed with 0.1 M HCl (2 × 5 mL) and brine (2 × 5 mL), dried over MgSO_4,_ and concentrated under reduced pressure. The oily residue was purified by chromatography on silica gel eluting with CH_2_Cl_2_/MeOH (95:5) to give a viscous oil which crystallized on standing. Trituration in Et_2_O gave the title compound as white crystals (2.42 g, 62%). M.p; 48 °C; IR (neat, cm^−1^) *ν* = 3529, 3350, 2926, 2854, 1765 (w), 1693 (s), 1437, 1404, 1358, 1338, 1288, 1193, 1157, 1126, 1064, 1045, 1018, 997, 904, 875, 852, 813, 717, 708; ^1^H NMR (300 MHz, CDCl_3_) *δ*: 6.51 (s, 2 H, OC*H*C=C*H*CO), 5.29 (s, 2 H, *H*COC*H*), 3.78–3.62 (m, 6H, NCH_2_C*H*_2_OC*H*_2_C*H*_2_OH), 3.57–3.52 (m, 2H, N*C*H_2_CH_2_O), 2.86 (s, 2 H, *H*CCON), 2.02 (br s, 1H, O*H*); ^13^C NMR (75 MHz, CDCl_3_) *δ*: 176.2 (C, 2CO), 136.4 (2CH, OCH*C*=*C*HCO), 80.9 (2CH, H*C*O*C*H), 72.2 (CH_2_, O*C*H_2_CH_2_OH), 66.9 (CH_2_, NCH_2_*C*H_2_O), 61.4 (CH_2_, OCH_2_*C*H_2_OH), 47.3 (2CH, *H*CCON), 38.4 (CH_2_, N*C*H_2_CH_2_O); MS (ESI+) *m/z* (%): 529.2 (100) [2M+Na]^+^, 276.1(55) [M+Na]^+^, 208.1(50) [M-C_4_H_4_O +Na]^+^. 

**Figure 2:**
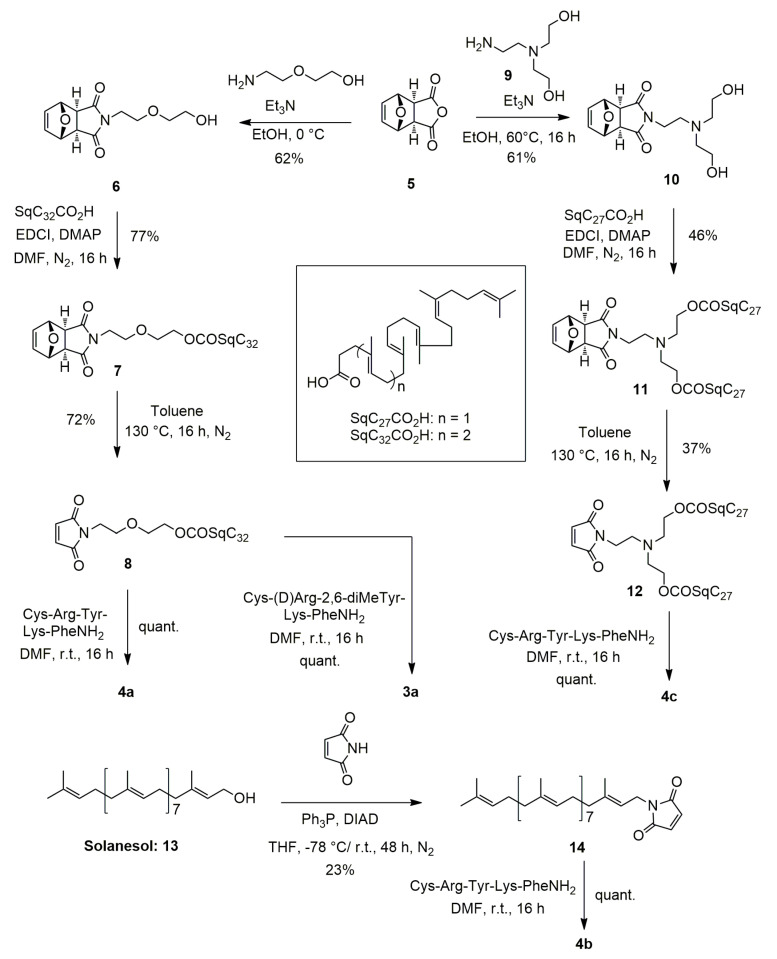
Synthetic scheme for obtaining bioconjugates **3a**, **4a**–**c**.

#### 2.3.3. Synthesis of 2-(2-{3,5-dioxo-10-oxa-4-azatricyclo [5.2.1.0^2,6^]dec-8-en-4-yl}ethoxy)ethyl (4E,8E,12E,16E,20E)-4,8,12,17,21,25-hexamethylhexacosa-4,8,12,16,20,24-hexaenoate (**7**) (Figure 2)

To a solution of squalene acetic acid (SqC_32_CO_2_H) (310 mg, 0.66 mmol) and alcohol **6** (202 mg, 0.80 mmol, 1.2 equiv.) in anhydrous Dimethylformamide (DMF) (8 mL) was added EDCI (138 mg, 0.72 mmol) and DMAP (15 mg, 0.13 mmol). The reaction mixture was stirred at room temperature, monitoring reaction advancement by TLC eluting with cyclohexane/AcOEt (2:1). After 48 h, the reaction mixture was concentrated under reduced pressure and the residue was taken up into saturated aqueous NH_4_Cl (10 mL) and extracted with ethyl acetate (3 × 100 mL). The combined organic layers were dried over MgSO_4_, filtered, and concentrated under reduced pressure. The crude product was purified by chromatography on silica gel eluting with cyclohexane/AcOEt (2:1) to give the title compound as a colorless oil (358 mg, 77%). IR (neat, cm^−1^) *ν* = 3749, 2926, 2848, 1700, 1602, 1397, 1141, 1025, 872; ^1^H NMR (300 MHz, CDCl_3_) *δ*: 6.51 (s, 2 H, OC*H*C=C*H*CO), 5.26 (s, 2 H, *H*COC*H*), 5.20–5.02 (m, 6 H, *H*C=C(Me)), 4.16 (t, *J* = 4.8 Hz, 2 H, OCH_2_C*H*_2_OCO), 3.72–3.57 (m, 6H, NC*H*_2_C*H*_2_OC*H*_2_CH_2_OCO), 2.85 (s, 2 H, *H*CCON), 2.45–2.35 (m, 2 H, CH_2_C*H*_2_CO_2_), 2.35–2.25 (m, 2 H, CH_2_C*H*_2_CO_2_), 2.15–1.95 (m, 20 H, =CC*H*_2_C*H*_2_), 1.68 (s, 3 H, (C*H*_3_)_2_C=), 1.60 (s, 15 H, C(C*H*_3_)CH_2_), 1.56 (s, 3 H, (C*H*_3_)_2_C=); ^13^C NMR (75 MHz, CDCl_3_) *δ*: 176.0 (2C, *C*ON*C*O), 173.3 (C, *C*O_2_), 136.5 (2CH, OCH*C*=*C*HCO), 135.0 (2C, =*C*(CH_3_)CH_2_), 134.8 (C, =*C*(CH_3_)CH_2_), 134.6 (C, =*C*(CH_3_)CH_2_), 133.1 (C, =*C*(CH_3_)CH_2_), 125.1 (CH, =*C*H(CH_2_)_2_), 124.4 (CH, =*C*H(CH_2_)_2_), 124.35 (CH, =*C*H(CH_2_)_2_), 124.3 (2CH, =*C*H(CH_2_)_2_), 124.2 (CH, =*C*H(CH_2_)_2_), 80.8 (2CH, H*C*O*C*H), 68.6 (CH_2_, NCH_2_CH_2_O*C*H_2_), 67.0 (CH_2_, N*C*H_2_*C*H_2_OCH_2_CH_2_), 63.3 (CH_2_, OCH_2_*C*H_2_OCO), 47.4 (2CH, *H*CCON), 39.7 (3CH_2_, =C(CH_3_)*C*H_2_CH_2_), 39.5 (CH_2_, =C(CH_3_)*C*H_2_CH_2_), 38.0 (CH_2_, N*C*H_2_CH_2_), 34.5 (CH_2_, O_2_CCH_2_*C*H_2_), 33.0 (CH_2_, O_2_C*C*H_2_CH_2_), 28.2 (2CH_2_, =CH*C*H_2_CH_2_), 26.9 (CH_2_, =CH*C*H_2_CH_2_), 26.7 (CH_2_, =CH*C*H_2_CH_2_), 26.6 (2CH_2_, =CH*C*H_2_CH_2_), 25.7 (CH_3,_ =C(*C*H_3_)_2_), 17.6 (CH_3,_ =C(*C*H_3_)_2_), 16.0 (4CH_3,_=C(*C*H_3_)CH_2_), 15.9 (CH_3,_ =C(*C*H_3_)CH_2_); MS (ESI+) *m/z* (%): 1429.3 (40)[2M+Na]^+^, 742.6 (35) [M+K]^+^, 726.6 (100) [M+Na]^+^, 704.6 (52) [M+H]^+^.

#### 2.3.4. Synthesis of 2-[2-(2,5-dioxo-2,5-dihydro-1H-pyrrol-1-yl)ethoxy]ethyl (4E,8E,12E,16E,20E)-4,8,12,17,21,25-hexamethylhexacosa-4,8,12,16,20,24-hexaenoate (**8**)

A solution of the above ester **7** (750 mg, 1.06 mmol) in anhydrous toluene (50 mL) was heated under reflux (oil bath, 130 °C) for 16 h. The reaction was monitored via TLC using cyclohexane/AcOEt 2:1 as eluant. The toluene was distilled under reduced pressure and crude product was purified by chromatography on silica gel eluting with ethyl acetate/cyclohexane (20/80) to give maleimide **8** as a colorless oil (487 mg, 72.3 %). IR (neat, cm^−1^) ν = 3749, 3669, 2973, 2906, 2327, 1699, 1602, 1394, 1251, 1037 (s), 803; ^1^H NMR (300 MHz, CDCl_3_) δ: 6.68 (s, 2 H, *H*C=C*H*), 5.12–5.05 (m, 6 H, *H*C=C(Me)), 4.14 (t, *J* = 3 Hz, 2 H, CH_2_C*H*_2_OCO), 3.71 (m, *J* = 3 Hz, 2 H, OCH_2_C*H*_2_), 3.62 (m, *J* = 6 Hz, 4 H, OC*H*_2_CH_2_), 2.38 (m, *J* = 3 Hz, 2 H, OCOC*H*_2_CH_2_), 2.26 (t, *J* = 6 Hz, 2 H, OCOCH_2_C*H*_2_), 2.04–1.97 (m, 20 H,=CC*H*_2_C*H*_2_), 1.65 (s, 3 H, (C*H*_3_)_2_C=), 1.58 (s, 18 H, C(C*H*_3_)CH_2_); ^13^C NMR (75 MHz, CDCl_3_) *δ*: 173.3 (C, *C*O_2_), 170.5 (2C, *C*ON*C*O), 135.0 (2C, =*C*(CH_3_)CH_2_), 134.8 (C, =*C*(CH_3_)CH_2_), 134.7 (=*C*(CH_3_)CH_2_), 134.1 (2CH, H*C*=*C*H), 133.1 (C, =*C*(CH_3_)CH_2_), 131.1 (C, =*C*(CH_3_)_2_), 125.1 (CH, =*C*HCH_2_CH_2_), 124.4 (CH, =*C*HCH_2_CH_2_), 124.3 (4CH, =*C*HCH_2_CH_2_), 68.5 (CH_2_, O*C*H_2_CH_2_OCO), 67.0 (CH_2_, O*C*H_2_CH_2_), 63.3 (CH_2_, OCH_2_*C*H_2_OCO), 39.7 (3CH_2_, =C(CH_3_)*C*H_2_CH_2_), 39.5 (CH_2_, =C(CH_3_)*C*H_2_CH_2_), 37.0 (CH_2_, N*C*H_2_CH_2_), 34.5 (CH_2_, O_2_CCH_2_*C*H_2_), 33.1 (CH_2_, O_2_C*C*H_2_CH_2_), 28.2 (2CH_2_, =CH*C*H_2_CH_2_), 26.7 (CH_2_, =CH*C*H_2_CH_2_), 26.6 (3CH_2_, =CH*C*H_2_CH_2_), 25.7 (CH_3_, =C(*C*H_3_)_2_), 17.6 (CH_3_, =C(*C*H_3_)_2_), 16.0 (4CH_3,_ =C(*C*H_3_)CH_2_), 15.9 (CH_3,_=C(*C*H_3_)CH_2_); MS (ESI+) *m/z* (%): 1293.0 (10) (2M+Na]^+^, 674.6 (20) [M+K]^+^, 658.6 (100) [M+Na]^+^, 636.6 (10) [M+H]^+^.

#### 2.3.5. Synthesis of Squalene Acetic Acid Conjugate of Cys-Arg-Tyr-Lys-PheNH_2_ (**4a**)

To a solution of squalene maleimide **8** (105.7 mg, 0.16 mmol) in anhydrous DMF (10 mL) was added 1.0 equiv. of the peptide **2b** (175.5 mg 0.16 mmol) and the reaction mixture was stirred 5 h at 20 °C. The mixture was concentrated under reduced pressure to the conjugate, which was used directly without further purification. IR (neat, cm^−1^) ν = 3600–2800, 2926, 1663, 1516, 1437, 1404, 1339, 1200, 1182, 1130, 839, 800, 723; ^1^H NMR (400 MHz, CD_3_OD) the presence of two diastereomers in 1:1 ratio induced splitting of most signals *δ*: 7.28–7.21 (m, 4H, H-2′_Phe_, H-3′_Phe_, H-5′_Phe_, H-6′_Phe_), 7.21–7.15 (m, 1H, H-4′_Phe_), 7.07 and 7.05 (2d, *J* = 7.6 Hz, 2 H, 2H, H-2′_Tyr_, H-6′_Tyr_), 6.71 and 6.69 (2d, *J* = 7.6 Hz, 2H, H-3′_Tyr_, H-5′_Tyr_), 5.18–5.06 (m, 6 H, *H*C=C(Me)), 4.59 (dd, *J* = 8.4 Hz, *J* = 5.6 Hz, 1H, H _αPhe_), 4.55–4.50 (m, 1H, H _αTyr_), 4.40–4.30 (m, 1H, H _αArg_), 4.20 (t, *J* = 6.8 Hz, 1H, H _αLys_), 4.30–4.05 (m, 1H, H _αCys_), 4.17–4.11 (m, 3H, OCH_2_C*H*_2_OCO, CH_2_SC*H*CH_2_CO), 3.75–3.60 (m, 6H, NC*H*_2_C*H*_2_OC*H*_2_CH_2_OCO), 3.47–3.39 (m, 0.5H, H _βCys_), 3.35–3.05 (m, 5.5H, H_βPhe_, H_δArg_, H_βCys_, CH_2_SCHC*H*_2_CO), 3.05–2.85 (m, 6H, H_βTyr_, H_βPhe_, H_εLys_), 2.81 (dd, *J* = 14.0 Hz, 9.0 Hz, 1H, H_βTyr_), 2.54 (dd, *J* = 18.8 Hz, 4.0 Hz, 1H, CH_2_SCHC*H*_2_CO), 2.39 (t, *J* = 7.4 Hz, 2 H, OCOC*H*_2_CH_2_), 2.26 (t, *J* = 7.8 Hz, 2 H, OCOCH_2_C*H*_2_), 2.15–1.90 (m, 20H, =CC*H*_2_C*H*_2_), 1.83–1.50 (m, 8H, H_βArg_, H_βLys_, H_γArg_, H_δLys_), 1.65 (s, 3 H, (C*H*_3_)_2_C=), 1.58 (s, 18 H, C(C*H*_3_)CH_2_), 1.40–1.25 (m, 2H, H _γLys_); ^13^C NMR (100 MHz, CD_3_OD) *δ*: 179.6 and 179.4 (C, SCH*C*ONCO), 176.3 (C, SCHCON*C*O), 175.8 (C, *C*ON_Phe_), 175.1 (C, Sq*C*O_2_), 173.6–173.0 (m, 3C, *C*ON_Tyr_, *C*ON_Arg_, *C*ON_Lys_), 169.0 and 168.8 (C, *C*ON_Cys_), 158.7 (C, N*C*=N_Arg_), 157.3 (C, C-4′_Tyr_), 138.3 (C, C-1′_Phe_), 135.9 (3C, =*C*(CH_3_)CH_2_), 135.8 (C, =*C*(CH_3_)CH_2_), 135.7 (2C, =*C*(CH_3_)CH_2_), 134.5 (C, =*C*(CH_3_)CH_2_), 132.0 (2CH, C-2′_Tyr_, C-6′_Tyr_), 130.3 (2CH, C-3′_Phe_, C-5′_Phe_), 129.5 (2CH, C-2′_Phe_, C-6′_Phe_), 128.7 and 128.6 (C, C-1′_Tyr_), 127.8 (CH, C-4′_Phe_), 126.3 (CH, =*C*H(CH_2_)_2_), 125.5 (4CH, =*C*H(CH_2_)_2_), 125.4 (CH, =*C*H(CH_2_)_2_), 116.3 (2CH, C-3′_Tyr_, C-5′_Tyr_), 69.6 (CH_2_, O*C*H_2_CH_2_OCO), 67.9 (CH_2_, NCH_2_*C*H_2_O), 64.5 (CH_2_, OCH_2_*C*H_2_OCO), 56.2 (CH, CH _αTyr_), 55.7 (CH, CH_αPhe_), 54.9 and 54.7 (2CH, CH_αArg_, CH_αLys_), 54.0 and 53.8 (CH, CH_αCys_), 41.9 (CH_2_, CH_2δArg_), 41.7 (CH, CH_2_S*C*HCH_2_CO), 40.8 (3CH_2_, =C(CH_3_)*C*H_2_CH_2_), 40.7 (CH_2_, =C(CH_3_)*C*H_2_CH_2_), 40.5 (CH_2_, CH_2_, CH_2εLys_), 39.5 (CH_2_, N*C*H_2_CH_2_), 38.7 (CH_2_, CH_2βPhe_), 37.9 (CH_2_, CH_2βTyr_), 36.7 and 36.6 (CH_2_, CH_2_SCHC*H*_2_CO), 35.7 (CH_2_, O_2_C*C*H_2_CH_2_), 34.6 and 34.2 (CH_2_, CH_2βCys_), 34.1 (CH_2_, O_2_CCH_2_*C*H_2_), 32.2 (CH_2_, CH_2βLys_), 30.1 (m, CH_2βArg_), 29.2 (2CH_2_, =CH*C*H_2_CH_2_), 27.9 (CH_2_, =CH*C*H_2_CH_2_), 27.8 (CH_2_, =CH*C*H_2_CH_2_), 27.7 (CH_2_, CH_2δLys_), 27.6 (CH_2_, =CH*C*H_2_CH_2_), 27.5 (CH_2_, =CH*C*H_2_CH_2_), 26.1 and 26.0 (CH_2_, CH_2γArg_), 25.9 (CH_3_, =C(*C*H_3_)_2_), 23.4 (CH_2_, CH_2γLys_), 17.6 (CH_3_, =C(*C*H_3_)_2_), 16.2 (4CH_3,_ =C(*C*H_3_)CH_2_), 16.0 (CH_3,_ =C(*C*H_3_)CH_2_); MS (ESI+) *m/z* (%): 1351.0 (5) [M^3+^-2H]^+^, 676.0(100) [M^3+^-H]^2+^, 451.1 (50) [M]^3+^.

#### 2.3.6. Synthesis of 1-[(2E,6E,10E,14E,18E,22E,26E,30E)-3,7,11,15,19,23,27,31,35-nonamethylhexatria-conta-2,6,10,14,18,22,26,30,34-nonaen-1-yl]-2,5-dihydro-1H-pyrrole-2,5-dione (**14**)

A solution of triphenylphosphine (263.0 mg, 1.0 mmol) in anhydrous Tetrahydrofuran (THF) (5 mL) was cooled to −78 °C (acetone/dry ice). Diisopropyl azodicarboxylate (22.0 mg, 1.1 mmol) was added dropwise. The mixture was stirred for 15 min and the temperature was risen to −20 °C. A solution of solanesol **13** (948 mg, 1.5 mmol) in THF (3 mL) was then added, followed with maleimide (97.0 mg, 1.0 mmol). The reaction mixture was stirred at 20 °C for 48 h and concentrated under reduced pressure. The residue was taken up into water and extracted with petroleum ether (3 × 80 mL). The combined organic layers were dried over MgSO_4_, filtered, and concentrated. The crude product was purified by chromatography on silica gel eluting with petroleum ether/Et_2_O (90:10) to leave the title compound as a colorless wax (184.3 mg, yield 23%). IR (neat, cm^−1^) ν = 2963, 2943, 2911, 2851, 1703, 1435, 1404, 1385, 1323, 1279, 1211, 1146, 1109, 997, 980, 962, 876, 835, 797, 773, 752, 692; ^1^H NMR (300 MHz, CDCl_3_) δ: 6.66 (s, 2 H, CO*H*C=C*H*CO), 5.20–5.04 (m, 9H, *H*C=C(Me)), 4.10 (d, *J* = 7.2 Hz, 2H, NC*H*_2_CH=), 2.11–1.98 (m, 32H, =CC*H*_2_C*H*_2_), 1.77 (s, 3H, (C*H*_3_)_2_C=), 1.68 (s, 3H, (C*H*_3_)_2_C=), 1.60 (s, 21H, C(C*H*_3_)CH_2_), 1.58 (s, 3H, C(C*H*_3_)CH_2_); ^13^C NMR (75 MHz, CDCl_3_) *δ*: 170.1 (2C, O*C*N*C*O), 142.4 (C, CH_2_(CH_3_)*C*=CHCH_2_N), 135.6 (C, CH_2_(CH_3_)*C*=CH), 135.1 (7C, CH_2_(CH_3_)*C*=CH), 134.3 (2CH, CO*C*H=*C*HCO), 131.34 (C, (CH_3_)_2_*C*=CH), 124.6–124.3 (7CH, (CH_3_)C=*C*HCH_2_), 123.8 (CH, (CH_3_)_2_*C*=CH), 118.1 (CH, (CH_3_)*C*=CHCH_2_N), 39.9 (7CH_2_, *C*H_2_(CH_3_)C=CH), 39.6 (CH_2_, *C*H_2_(CH_3_)C=CH), 35.8 (CH_2_, CH_2_N), 26.9 (7CH_2_, (CH_3_)C=CH*C*H_2_), 26.4 (CH_2_, (CH_3_)C=CH*C*H_2_), 25.8 (CH_3_, (*C*H_3_)_2_C=CH), 16.4 (CH_3_, (*C*H_3_)_2_C=CH), 16.2 (7CH_3_, CH_2_(*C*H_3_)C=); MS (ESI+) *m/z* (%): 732.6 (100) [M+Na]^+^.

#### 2.3.7. Synthesis of Solanesol Conjugate of Cys-Arg-Tyr-Lys-Phe-NH_2_ (**4b**)

To a stirred solution of the above solanesol maleimide **14** (36.6 mg, 0.051 mmol) in anhydrous DMF (2 mL) was added 1.0 equiv. of the peptide **2b** (54.4 mg, 0.051 mmol) and the reaction was left 16 h under agitation overnight. The mixture was concentrated under reduced pressure to give the conjugate, which was used directly without further purification. IR (neat, cm^−1^) *ν* = 3600–2800, 2965, 2918, 2853, 1668, 1636, 1539, 1516, 1435, 1398, 1202, 1182, 1134, 837, 800, 721, 696; ^1^H NMR (400 MHz, DMSO) the presence of two diastereomers in 1:1 ratio induced splitting of some signals *δ*: 9.15 and 9.14 (2s, 1H, PhO*H*), 8.62 (br s, 1H), 8.45–8.25 (m, 2H, N*H_3_*), 8.11 (d, *J* = 7.6 Hz, 1H, CON*H*_Lys_), 8.00 (dd, *J* = 11.2 Hz, *J* = 8.8 Hz, 1H, CON*H*_Tyr or_ CON*H*_Phe_), 7.81 (d, *J* = 8.0 Hz, 1H, CON*H*_Tyr or_ CON*H*_Phe_), 7.9–7.5 (m, 4 H, *H*_2_NC=N*H*_2_), 7.42 (s, 1H, CON*H*_2_), 7.27–6.90 (m, 7H, H-2′ to H-6′_Phe,_ CON*H*_Tyr_),7.08 (s, 1H, CON*H*_2_), 6.99 and 6.98 (2d, *J* = 8.4 Hz, 2H, H-2′_Tyr_, H-6′_Tyr_), 6.61 and 6.60 (2d, *J* = 8.4 Hz, 2H, H-3′_Tyr_, H-5′_Tyr_), 5.06 (m, 9 H, *H*C=C(Me)), 4.50–4.40 (m, 2H, H _αTyr_, H _αPhe_), 4.37–4.29 (m, 1H, H_αArg_), 4.20–4.15 (m, 1H, H_αLys_), 4.10 (dd, *J* = 9.3 Hz, *J* = 4.8 Hz, 0.5 H, H_αCys_), 4.00 (dd, *J* = 8.8 Hz, *J* = 4.4 Hz, 0.5 H, H_αCys_), 3.97 (d, *J* = 6.8 Hz, 2H, NC*H*_2_CH=), 3.30–3.22 (m, CH_2_, (m, 1H, H _βCys_), ), 3.20–3.07 (m, 2H, CH_2_SCHC*H*_2_CON), 3.10–3.04 (m, 2H, H _δArg_), 3.01 (dd, *J* = 13.6 Hz,, *J* = 5.2 Hz, 1H, H_βTyr_), 2.98–2.85 (m, 1H, H_βCys_), 2.84 (dd, *J* = 13.6 Hz,, *J* = 8.4 Hz, 1H, H_βTyr_), 2.77–2.60 (m, 2H, H_εLys),_ 2.55.2.45 (m, 2H, CH_2_SCHC*H*_2_CON)_,_ 2.06–1.89 (m, 32 H, =CC*H*_2_C*H*_2_), 1.69 (s, 3H, (C*H*_3_)_2_C=), 1.63 (s, 3H, (C*H*_3_)_2_C=), 1.54 (s, 24H, C(C*H*_3_)CH_2_), 1.55–1.40 (m, 8H, H_βArg_, H_βLys_, H_γArg_, H_δLys_), 1.28–1.14 (m, 2H, H_γLys_); ^13^C NMR (75 MHz, CDCl_3_) *δ*, 176.7 and 176.4 (C, SCH*C*ONCO), 174.4 (C, SCHCON*C*O), 172.6 (C, *C*ON _Phe_), 170.9 (2C, *C*ON _Tyr_, *C*ON _Lys_), 170.4 (C, *C*ON_Arg_,), 156.7 (C, N*C*=N_Arg_), 155.7 (C, C-4′_Tyr_), 139.7 and 139.6 (C, NCH_2_CH=*C*(Me)CH_2_), 137.6 (C, C-1′_Phe_), 134.6 (C, CH_2_(CH_3_)*C*=CH), 134.5 (C, CH_2_(CH_3_)*C*=CH), 134.3–134.0 (m, 7C, CH_2_(CH_3_)*C*=CH), 130.5 (C, (CH_3_)_2_*C*=CH), 130.0 (2CH, C-2′_Tyr,_ C-6′_Tyr_), 129.1 (2CH, C-3′_Phe_, C-5′_Phe_), 127.9 (2CH, C-2′_Phe_, C-6′_Phe_), 127.4 (C, C-1′_Tyr_), 126.2 (CH, C-4′_Phe_), 124.1 (2CH, CH_2_(CH_3_)C=*C*H), 123.9 (5 CH, CH_2_(CH_3_)C=*C*H), 123.5 (CH, CH_2_(CH_3_)C=*C*H), 117.5 (CH, NCH_2_*C*H=), 114.8 (2CH, C-3′_Tyr_, C-5′_Tyr_), 53.8 (CH, CH_αTyr_ or CH_αPhe_), 53.4 (CH, CH_αPhe_ or CH_αTyr_), 52.7 (CH, CH_αLys_), 52.3 (2CH, CH_αArg_, CH_αCys_), 40.4 (CH_2_, CH_2δArg_), 39.8–38.5 (10CH_2_, CH_2εLys_, CH_2_S*C*HCH_2_CO and 8 *C*H_2_(CH_3_)C=CH), 37.5 (CH_2_, CH _βPhe_), 36.6 (CH_2_, *C*H_2 βTyr_), 36.3 (CH_2_, CH_2_N), 35.7 and 35.6 (CH_2_, CH_2_SCH*C*H_2_CO), 31.3 (CH_2_, CH_2βLys_), 29.4 (CH_2_, CH_2βArg_), 26.6–24.5 (m, 11CH_2_, (CH_3_)C=CH*C*H_2_CH_2_, CH_2δLys_, CH_2βCys_,), 25.4 (CH_3_, (*C*H_3_)_2_C=), 24.8 (CH_2γLys_), 22.0 (CH_2_, CH_2 γLys_), 17.4 (CH_3_, (*C*H_3_)_2_C=), 16.0 (CH_3_, CH_2_(*C*H_3_)C=), 15.7 (7CH_3_, CH_2_(*C*H_3_)C=) (several carbons were hidden by the solvent picks); MS (ESI+) *m/z* (%): 1424.9 (10) [M+H]^+^, 713.1(100) [M+2H]^2+^.

#### 2.3.8. Synthesis of 4-{2-[bis(2-hydroxyethyl)amino]ethyl}-10-oxa-4-azatricyclo [5.2.1.0^2,6^]dec-8-ene-3,5-dione (**10**)

To a stirred solution of 4,10-dioxatricyclo [5.2.1.0^2,6^]dec-8-ene-3,5-dione (5) [22], (390 mg, 2.35 mmol) in anhydrous EtOH (2.5 mL) was added a solution of 2,2′-[(2-aminoethyl)imino]diethanol (522 mg, 3.52 mmol, 1.5 equiv.) in EtOH (1 mL) followed by triethylamine (261 mg, 2.58 mmol). The reaction mixture was stirred at 60 °C for 16 h. The advancement of the reaction was monitored by TLC eluting with CH_2_Cl_2_/MeOH (95:5) as eluant. The reaction mixture was concentrated under reduced pressure and the crude product was purified using chromatography on silica gel eluting with CH_2_Cl_2_/MeOH (95:5 to 90:10) to give the title compound as a viscous colorless oil (426 mg, 61.4% yield). IR (neat, cm^−1^) ν = 3600–3200, 2949, 2880, 1771, 1690, 1435, 1402, 1335, 1314, 1190, 1152, 1094, 1072, 1043, 1018, 914, 878, 853, 824, 806, 721, 650; ^1^H NMR (300 MHz, CDCl_3_) *δ*: 6.43 (t, *J* = 0.9 Hz, 2H, *H*C=C*H*), 5.19 (t, *J* = 0.9 Hz, 2 H, *H*COC*H*), 3.50 (t, *J* = 5.7 Hz, 2 H, (CO)_2_NC*H*_2_CH_2_), 3.44 (t, *J* = 5.1 Hz, 4 H, NCH_2_C*H*_2_OH), 3.07 (br s, 2 H, CH_2_CH_2_O*H*), 2.82 (s, 2 H, *H*CCON), 2.65 (t, *J* = 5.7 Hz, 2 H, (CO)_2_NCH_2_C*H*_2_), 2.55 (t, *J* = 5.1 Hz, 4 H, OHCH_2_C*H*_2_); ^13^C NMR (75 MHz, CDCl_3_) *δ*: 177.1 (2C, *C*ON*C*O), 136.5 (2CH, H*C*=*C*H), 81.0 (2CH, H*C*O*C*H), 59.8 (2CH_2_, N(CH_2_*C*H_2_OH)_2_), 57.1 (2CH_2_, N(*C*H_2_CH_2_OH)_2_), 53.0 (CH_2_, *C*H_2_N(CH_2_CH_2_OH)_2_), 47.6 (2CH, *H*CCON), 37.9 (CH2, (CO)_2_N*C*H_2_CH_2_N); MS (ESI+) *m/z* (%): 297.2 (100) [M+H]^+,^ 229.2 (65) [M-C_4_H_4_O +H]^+^.

#### 2.3.9. Synthesis of 2-{[2-(2,5-dioxo-2,5-dihydro-1H-pyrrol-1-yl)ethyl](2-{[(4E,8E,12E,16E)-4,8,12,17,21-pentamethyldocosa-4,8,12,16,20-pentaenoyl]oxy}ethyl) amino}ethyl (4E,8E,12E,16E)-4,8,12,17,21-pentamethyldocosa-4,8,12,16,20-pentaenoate (**12**)

To a solution of diol **10** (221 mg, 0.74 mmol, 1 equiv.) and SqC_27_CO_2_H (679 mg, 1.69 mmol, 2.3 equiv.) in anhydrous DMF (10 mL) were sequentially added DMAP (20 mg, 0.16 mmol) and EDCI (352 mg, 1.83 mmol, 2.5 equiv.). The reaction mixture was stirred at room temperature for the 16 h. The advance of the reaction was monitored by TLC using petroleum ether/AcOEt (80:20) as eluant. The reaction mixture was concentrated under reduced pressure and the residue was taken into water (3 mL) and extracted with petroleum ether (3 × 80 mL). The combined organic layers were dried over MgSO_4_ and concentrated under reduced pressure. The crude product was purified by chromatography on silica gel eluting with petroleum ether/AcOEt (20:80) to give the corresponding diester **11** as a viscous colorless oil (359 mg, 45.9% yield). The obtained product was taken up into anhydrous toluene (30 mL) and reflux for 16 h (oil bath, 130 °C). After cooling, the reaction mixture was concentrated under reduced pressure and the crude product was purified by chromatography on silica gel eluting with petroleum ether/AcOEt (2:1) to provide maleimide **12** as a viscous colorless oil (124 mg, 37% yield). IR (neat, cm^−1^) ν = 2961, 2914, 2853, 1736, 1709, 1437, 1406, 1383, 1290, 1155, 1040, 980, 826, 696; ^1^H NMR (300 MHz, CDCl_3_) *δ*: 6.67 (s, 2 H, *H*C=C*H*), 5.15–5.07 (m, 10 H, *H*C=C(Me)), 4.07 (t, *J* = 6.0 Hz, 4H, COOC*H*_2_CH_2_), 3.59 (t, *J* = 6.5 Hz, 2H, (CO)_2_NC*H*_2_CH_2_), 2.83–2.75 (m, 6H, NC*H*_2_CH_2_), 2.41–2.35 (m, 4H, O_2_CC*H*_2_CH_2_), 2.31–2.24 (m, 4H, O_2_CCH_2_C*H*_2_), 2.15–1.91 (m, 32H, =C(CH_3_)C*H*_2_C*H*_2_), 1.67 (s, 6H, (C*H*_3_)_2_C=), 1.60 (s, 30H, C(C*H*_3_)CH_2_); ^13^C NMR (75 MHz, CDCl_3_) δ: 173.4 (2C, *C*O_2_), 170.8 (2C, O*C*N*C*O), 135.3 (2C,=*C*(CH_3_)CH_2_CH_2_), 135.1 (2C,=*C*(CH_3_)CH_2_CH_2_), 135.0 (2C,=*C*(CH_3_)CH_2_CH_2_), 134.2 (2CH, CO*C*H=*C*HCO), 133.4 (2C,=*C*(CH_3_)CH_2_CH_2_), 131.4 (2C,=*C*(CH_3_)_2_), 125.3 (2CH, (CH_3_)C=*C*H), 124.6 (4CH, (CH_3_)C=*C*H), 124.4 (4CH, (CH_3_)C=*C*H), 62.6 (2CH_2_, (SqCO_2_*C*H_2_*C*H_2_)_2_N), 52.9 (2CH_2_, (SqCO_2_CH_2_*C*H_2_)_2_NCH_2_CH_2_N), 52.6 (CH_2_, (SqCO_2_CH_2_CH_2_)_2_N*C*H_2_CH_2_N), 39.9 (4CH_2_, =C(CH_3_)*C*H_2_CH_2_), 39.7 (4CH_2_, =C(CH_3_)*C*H_2_CH_2_), 36.2 (CH_2_, *C*H_2_NCOCH=), 34.7 (2CH_2_, O_2_CCH_2_*C*H_2_), 33.3 (2CH_2,_ O_2_C*C*H_2_CH_2_), 28.4 (6CH_2_, (CH_3_)C=CH*C*H_2_CH_2_), 26.9 (6CH_2_, (CH_3_)C=CH*C*H_2_CH_2_), 25.8, (2CH_3_,=*C*(CH_3_)_2_), 17.8 (2CH_3_, CH_2_(*C*H_3_)C=), 16.2 (6CH_3_, CH_2_(*C*H_3_)C=), 16.1 (2CH_3_, CH_2_(*C*H_3_)C=), 16.0 (2CH_3_, CH_2_(*C*H_3_)C=); MS (ESI+) *m/z* (%): 993.9 (100) [M+H]^+^.

#### 2.3.10. Synthesis of Bis-Squalene Conjugate of Cys-Arg-Tyr-Lys-PheNH_2_ (**4c**)

To a solution of maleimide **15** (48.3 mg, 0.048 mmol) in anhydrous DMF (5 mL) was added 51.4 mg (0.048 mmol) of peptide **2b** and the reaction was left 16 h under agitation overnight. The mixture was concentrated under reduced pressure to leave the conjugate, which was used directly without further purification. IR (neat, cm^−1^) ν = 3600–2800, 2924, 2850, 1690, 1664, 1635, 1558, 1541, 1516, 1436, 1404, 1382, 1338, 1199, 1180, 1132, 837, 800, 721, 698; ^1^H NMR (400 MHz, CD_3_OD) the presence of two diastereomers in 1:1 ratio induced splitting of some signals *δ*: 7.30–7.22 (m, 4H, H-2 _Phe_’, H-3′ _Phe_, H-5′_Phe_, H-6′_Phe_), 7.22–7.15 (m, 1H, H-4′_Phe_,), 7.07 and 7.05 (2d, *J* = 6.3 Hz, 2H, H-2′_Tyr_ and H-6′_Tyr_), 6.71 and 6.69 (2d, *J* = 6.3 Hz, 2H, H-3′_Tyr_ and H-5′_Tyr_), 5.25–5.05 (m, 10 H, *H*C=C(Me)), 4.59 (dd, *J* = 7.6 Hz, *J* = 6.4 Hz, 1H, H_αPhe_), 4.58–4.52 (m, 1H, H_αTyr_), 4.41–4.33 (m, 1H, H_αArg_), 4.31 (dd, *J* = 8.1 Hz, *J* = 5.2 Hz, 0.5H, H_αCys_), 4.27–4.18 (m, 1.5H, H_αCys_, H _αLys_), 4.15–3.98 (m, 5H, SC*H*CH_2_CONCO, N(CH_2_C*H*_2_OCOSq)_2_), 4.05–3.98 (m, 0.5H, C*H*SCH_2_CON), 3.59 (t, *J* = 6.2 Hz, 2H, (CO)_2_NC*H*_2_CH_2_), 3.45 (dd, *J* = 14.4 Hz, 6.4 Hz, 0.5H, H_βcys_), 3.34–3.25 (m, 1H, H_βCys_), 3.24–3.12 (m, 4, CHSC*H*_2_CON, H _δArg_, H _βPhe_), 3.10 (dd, 14.8 Hz, 7.6 Hz, 0.5 H, H _βCys_), 3.02–2.90 (m, 2H, H _βPhe,_ H _βTyr_), 2.88 (t, *J* = 7.6 Hz, 2H, H _εLys_), 2.87–2.75 (m, 7H, H_βTyr_, C*H*_2_N(C*H*_2_C*H*_2_OCOSq)_2_), 2.58–2.48 (m, 1H, CHSC*H*_2_CON), 2.41 (t, *J* = 7.4 Hz, 4H, O_2_CC*H*_2_CH_2_), 2.67 (t, *J* = 7.4 Hz, 4H, O_2_CCH_2_C*H*_2_), 2.15–1.95 (m, 32 H, =C(CH_3_)C*H*_2_C*H*_2_), 1.85–1.50 (m, 8H, CH_2βLys_, CH_2δLys_, CH_2βArg_, CH_2 γArg_), 1.67 (s, 6H, (C*H*_3_)_2_C=), 1.62–1.58 (m, 30H, C(C*H*_3_)CH_2_), 1.37–1.28 (m, 2H, CH_2γLys_); ^13^C NMR (100 MHz, CD_3_OD) the presence of two diastereomers in 1:1 ratio induced splitting of some signals *δ*: 179.9 and 179.5 (C, SCH*C*ONCO), 176.3 (C, SCHCON*C*O), 175.7 (C, *C*ON_Phe_), 175.0 (2C, Sq*C*O_2_), 173.6–172.9 (m, 3C, *C*ON_Tyr_, *C*ON_Arg_, *C*ON_Lys_), 169.0 and 168.9 (C, *C*O_Cys_), 158.7 (C, N*C*=N_Arg_), 157.3 (C, C-4′_Tyr_), 138.3 (C, C-1′_Phe_), 136.0 (2C,=*C*(CH_3_)CH_2_CH_2_), 135.9 (4C,=*C*(CH_3_)CH_2_CH_2_), 134.5 (2C,=*C*(CH_3_)CH_2_CH_2_), 132.0, (2C, =*C*(CH_3_)_2_), 131.4 (2CH, C-2′_Tyr,_ C-6′_Tyr_), 130.4 (2CH, C-3′_Phe_, C-5′_Phe_), 129.5 (2CH, C-2′_Phe_, C-6′_Phe_), 128.8 and 128.7 (C, C-1′_Tyr_), 127.8 (CH, C-4′_Phe_), 126.3 (2CH, (CH_3_)C=*C*H), 125.7 (2CH, (CH_3_)C=*C*H), 125.6 (2CH, (CH_3_)C=*C*H), 125.4 (4CH, (CH_3_)C=*C*H), 116.4 (2CH, C-3′_Tyr,_ C-5′_Tyr_), 63.9 (2CH_2_, (SqCO_2_*C*H_2_CH_2_)_2_N), 56.1 (CH, CH_αTyr_), 55.6 (CH, CH_αPhe_), 54.9–53.8 (m, 4CH, CH_αArg,_ CH_αLys,_ CH_αCys_), 53.1 (2CH_2_, (SqCO_2_CH_2_*C*H_2_)_2_NCH_2_), 52.7 (CH_2_, (SqCO_2_CH_2_CH_2_)_2_N*C*H_2_CH_2_N), 41.9 (CH_2_, CH_2δArg_), 40.9–40.5 (8CH_2_, 1CH, =C(CH_3_)*C*H_2_CH_2,_ OCCH_2_*C*HS), 40.5 (CH_2_, CH_2εLys_), 38.9 (CH_2_, CH_2 βPhe_), 38.2–37.9 (2CH_2_, *C*H_2_N(CO)_2_, CH_2βTyr_), 36.7 (CH_2_, CH_2_SCH*C*H_2_CO), 35.8 (2CH_2,_ O_2_CCH_2_*C*H_2_), 34.8 and 34.4 (CH_2,_ CH_2βCys_), 34.2 (2CH_2,_ O_2_C*C*H_2_CH_2_), 32.3 (CH_2_, CH_2βLys_), 30.2 (CH_2_, CH_2βArg_), 29.2 (6CH_2_, (CH_3_)C=CH*C*H_2_CH_2_), 28.0 (CH_2_, CH_2δLys_), 27.8–27.6 (6CH_2_, (CH_3_)C=CH*C*H_2_CH_2_), 26.1 (CH_2_, CH_2γArg_), 25.9 (2CH_3_, =*C*(CH_3_)_2_), 23.4 (CH_2_, CH_2γLys_), 17.8 (2CH_3_, =*C*(CH_3_)CH_2_), 16.3–16.1 (8CH_3_, =*C*(CH_3_)CH_2_); MS (ESI+) *m/z* (%):1708.2 (5) [M−2H]^+^, 854.1 (70) [M−H]^2+^, 570.4 (100) [M]^3+^.

#### 2.3.11. Synthesis of Squalene Acetic Acid Conjugate of Cys-D-Arg-diMeTyr-Lys-Phe-NH_2_ (**3a**)

To a solution of squalene maleimide **8** (29.8 mg, 0.047 mmol) in anhydrous DMF (5 mL) was added 1.0 equiv. of the peptide **2a** (50.8 mg, 0.047 mmol) and the reaction mixture was stirred 5 h at 20 °C. The mixture was concentrated under reduced pressure to the conjugate, which was used directly without further purification. IR (neat, cm^−1^) ν = 3600–2800, 2926, 1663, 1533, 1431, 1400, 1312, 1200, 1130, 1028, 835, 800, 721, 698; ^1^H NMR (400 MHz, CD_3_OD) the presence of two diastereomers in 1:1 ratio induced splitting of most signals *δ*: 7.31–7.23 (m, 4H, H-2′_Phe_, H-3′_Phe_, H-5′_Phe_, H-6′_Phe_), 7.22–7.16 (m, 1H, H-4′_Phe_), 6.44 (s, 2H, H-3′_Tyr_, H-5′_Tyr_), 5.20–5.05 (m, 6H, *H*C=C(Me)), 4.68 and 4.66 (2dd, *J* = 6.8 Hz, *J* = 3.6 Hz, 1H, H_αMeTyr_), 4.55 and-4.53 (2dd, *J* = 6.0 Hz, *J* = 2.8 Hz, 1H, H_αPhe_), 4.35–4.24 (m, 2.5 H*,* H_αArg,_ H_αLys,_ 0.5H_αCys_), 4.20 (dd, *J* = 7.6 Hz, *J* = 6.0 Hz, 0.5H, H_αCys_), 4.13 (t, *J* = 5.2 Hz, 2H, OCH_2_C*H*_2_OCO), 3.73–3.61 (m, 6H, NC*H*_2_C*H*_2_OC*H*_2_CH_2_OCO), 3.46 (dd, *J* = 14.8 Hz, *J* = 6.4 Hz, 0.5H, H_βCys_), 3.38–3.32 (m, 0.5H, H_βCys_), 3.35–3.05 (m, 7H, 1H_βCys_, 2H_δArg_, 1H_βPhe_, 1H_βMeTyr_, CH_2_SC*H*C*H*_2_CO), 2.99 (ddd, *J* = 11.6 Hz, *J* = 8.6 Hz, *J* = 2.0 Hz, 1H, 1H_βPhe_), 2.91 (t, *J* = 7.6 Hz, 2H, 1H _βLys_), 2.53 (dd, *J* = 18.4 Hz, *J* = 4.8 Hz, 1H, CH_2_SCHC*H*_2_CO), 2.41 (t, *J* = 7.4 Hz, 2 H, OCOC*H*_2_CH_2_), 2.30–2.24 (m, 8H, OCOC*H*_2_CH_2,_ C*H*_3_Ar), 2.15–1.93 (m, 20 H, =C(CH_3_)C*H*_2_C*H*_2_), 1.84–1.70 (m, 1H, CH_2βArg_)_,_ 1.68 (s, 3 H, (C*H*_3_)_2_C=), 1.70–1.52 1.60 (m, 16 H, =C(C*H*_3_)CH_2_, CH_2βLys,_ CH_2 δLys_), 1.50–1.26 (m, 4H, CH_2γLys,_ CH_2γArg_); ^13^C NMR (101 MHz, MeOD) *δ* 179.5 and 179.2 (C, SCH*C*ONCO), 176.1, (C, SCHCON*C*O), 175.8 (C, *C*ON_Phe_), 175.0 (C, Sq*C*O_2_), 173.4–173.0 (3C, *C*ON_Tyr_, *C*ON_Arg_, *C*ON_Lys_), 169.2 and 169.1 (C, *C*ON_Cys_), 163.1 (C, q, *J*_CF_ = 34.5 Hz, *C*O_2_CF_3_), 158.7 (C, N*C*=N_Arg_), 156.4 (C, C-4′_Tyr_), 139.7 (C, C-1′_Tyr_), 138.3 (C, C-1′_Phe_), 136.0 (2C, =*C*(CH_3_)CH_2_CH_2_), 135.8 (C, =*C*(CH_3_)CH_2_CH_2_), 135.7 (C, =*C*(CH_3_)CH_2_CH_2_), 134.5 (C, =*C*(CH_3_)CH_2_CH_2_), 132.0 (C, =*C*(CH_3_)_2_), 130.4 (2CH, C-3′_Phe_, C-5′_Phe_), 129.5 (2CH, C-2′_Phe_, C-6′_Phe_), 127.8 (CH, C-4′_Phe_), 126.3 (2CH, (CH_3_)C=*C*H), 126.1 (2C, C-2′_Tyr,_ C-6′_Tyr_), 125.6 (3CH, (CH_3_)C=*C*H), 125.5 (CH, (CH_3_)C=*C*H), 116.2 (2CH, C-3′_Tyr,_ C-5′_Tyr_), 69.7 (CH_2_, O*C*H_2_CH_2_OCO), 67.9 (CH_2_, NCH_2_*C*H_2_O), 64.5 (CH_2_, OCH_2_*C*H_2_OCO), 56.0 (CH, CH_αPhe_), 55.10 and 55.0 (CH, CH_αLys_ or CH_αArg_), 54.5 (CH, CH_αLys_ or CH_αArg_), 54.4 and 54.3 (CH, CH_αTyr_), 54.1 and 53.8 (CH, CH_αCys_), 42.0 (CH_2_, CH_2δArg_), 40.8 (3CH_2_, =C(CH_3_)*C*H_2_CH_2_), 40.7 (CH_2_, =C(CH_3_)*C*H_2_CH_2_), 40.5 (CH_2_, CH_2 εLys_), 39.5 and 39.4 (CH_2_, N*C*H_2_CH_2_), 38.7 (CH_2_, CH_2 βMeTyr_), 36.5 and 36.4 (CH_2_, CH_2_SCHC*H*_2_CO), 35.7 (CH_2_, O_2_CCH_2_*C*H_2_), 34.5 and 34.0 (CH_2_, CH_2βCys_), 34.1 (CH_2_, O_2_C*C*H_2_CH_2_), 32.4 (CH_2_, CH_2βArg_), 31.8 (CH_2_, CH_2βPhe_), 29.5 and 29.4 (CH_2_, CH_2βLys_), 29.2 (2CH_2_, =CH*C*H_2_CH_2_), 28.0 (CH_2_, CH_2δLys_), 27.8 (CH_2_, =C(CH_3_)C*H*_2_C*H*_2_), 27.7 (CH_2_, =C(CH_3_)C*H*_2_C*H*_2_), 27.6 (CH_2_, =C(CH_3_)C*H*_2_C*H*_2_), 27.5 (CH_2_, =C(CH_3_)C*H*_2_C*H*_2_), 25.9 (CH and CH_2_, =C(*C*H_3_)_2_, CH_2γArg_), 23.5 (CH_2_, CH_2γLys_), 20.7 (2CH_3_, CH_3 MeTyr_), 17.6 (CH_3_, =C(*C*H_3_)_2_), 17.8 (CH_3_, =C(*C*H_3_)CH_2_), 16.2 (CH_3_, =C(*C*H_3_)CH_2_), 16.0 (CH_3_, =C(*C*H_3_)CH_2_), The *C*HS of maleimide was not detected; MS (ESI+) *m/z* (%): 1378.9 (5) [M−2H]^+^, 690.0 (100) [M−H]^2+^, 460.3 (45) [M]^3+^.

### 2.4. Nanoparticle Obtention and Characterisation

SqCsA, SqCsA/**4a**, **4b** and **4c** nanoparticles (NPs) were obtained by the nanoprecipitation technique. Briefly, SqCsA, **4a**, **4b** and **4c** (Figure 1) were dissolved in absolute ethanol to a concentration of 6 mg/mL. Then, the organic solutions containing SqCsA and either **4a**, **4b** or **4c** were mixed, respectively, at 4 ratios (*v*/*v*): 98:2, 95:5, 90:10 and 75:25. The mixture was then added dropwise under vigorous stirring to 1 mL of a 5% (*w*/*v*) dextrose solution, and NPs were formed spontaneously without using any surfactant. After solvent evaporation using a Rotavapor (80 rpm, 40 °C, 43 mbar), aqueous suspensions of SqCsA/**4a**, **4b** or **4c** NPs were obtained at a final concentration of 2 mg/mL. The mean particle size, polydispersity index and zeta potential were all measured using a Zetasizer Nano ZS (173° scattering angle, 25 °C, Malvern). The measurements were performed after dilution of the NPs (1/20 *v*/*v*) in Milli-Q water (size) and NaCl 1 mM (zeta) and were carried out in triplicate. Colloidal stability was assessed by measuring the NPs mean diameter and size distribution over a period of 96 h or 30 days and under different storage conditions: 4 °C and room temperature (RT).

### 2.5. Morphology by CryoTEM

The morphology of multidrugs NPs composed of SqCsA/**4a**, **4b** or **4c** was observed by cryogenic transmission electron microscopy (cryoTEM) as previously published [20]. Briefly, a few drops of the NPs suspension (2 mg/mL) were placed on EM grids covered with a holey carbon film (Quantifoil R7/2) previously treated with a plasma glow discharge. The excess liquid was blotted, and the remaining thin film was quickly frozen in liquid ethane at cryogenic temperature using a Vitrobot (Thermo Fisher Scientific, Waltham, MA, USA). CryoTEM images were acquired on a JEOL 2010 FEG microscope (JEOL, Tokyo, Japan) operated at 200 kV and low temperature (−180 °C) using a Gatan camera (Gatan, Pleasanton, CA, USA).

### 2.6. X-ray Photoelectron Spectroscopy (XPS)

XPS was used to determine the chemical composition of the NPs surface using a ThermoElectron ESCALAB 250 Xi spectrometer (Thermo Fisher Scientific, Waltham, MA, USA), with a monochromatised Al Kα radiation (1486.6 eV). The analyzer pass energy was 100 eV for survey spectra and 20 eV for high resolution spectra. Core levels were analyzed for O 1s, C 1s, F 1s, N 1s, and S 2p. The photoelectron takeoff angle (angle of the surface with the direction in which the photoelectrons are analyzed) was 90°. Spectra were calibrated using C-C, C-H bonds at 285 eV. The curve fitting of the spectra was carried out with the Thermo Electron software (version 5.9925, Thermo Fisher Scientific, Waltham, MA, USA). 

Three samples were analyzed by XPS: SqCsA, SqCsA/**3a** 95:5 and 75:25. The nanoparticles in solution were freeze-dried and the resulting powder was attached to the sample holder with carbon tape.

### 2.7. Cell Culture

Immortalized Mouse Cardiac Endothelial Cells (MCEC) and rat cardiomyoblasts (H9c2) cells were cultured, respectively, in DMEM supplemented with 5% FBS, 1% penicillin–streptomycin, and 1% HEPES and DMEM supplemented with 10% FBS and 1% penicillin–streptomycin. Cells were maintained in a humid atmosphere with 5% CO_2_ at 37 °C.

### 2.8. Cytotoxicity of SqCsA/***4a***, ***4b*** or ***4c*** NPs

The cytotoxic activity of SqCsA/**4a**, **4b** and **4c** NPs was evaluated on MCEC and H9c2 cells using the 3-(4,5-dimethylthiazol-2-yl)-2,5-diphenyltetrazolium bromide (MTT) test. Cells were seeded in 96-well plates at 6000 cells per well (MCEC) and 10,000 cells per well (H9c2) and pre-incubated for 24 h at 37 °C in a 5% CO_2_ humidified incubator. Subsequently, cells were incubated with concentrations of SqCsA/**4a**, **4b** and **4c** NPs ranging from 1.2 to 60 µg/mL and the equivalent concentration of either the free bioconjugate (**4a**, **4b** or **4c**, dissolved in ethanol) or the free peptide (**2b**, dissolved in water) for 24 h. At the end of incubation, 20 µL of a MTT solution (5 mg/mL in PBS) was added to each well, and plates were incubated for 2 h at 37 °C. Thereafter, the culture medium was discarded, and 200 µL of DMSO were used in each well to dissolve formazan crystals. Plates were stirred for 10 min on a plate shaker, and the absorbance was measured at 570 nm using an ELISA plate reader. Cell viability was expressed as a percentage of the untreated cells (control wells).

### 2.9. Cell Uptake of Multidrug Nanoparticles

Cell uptake was investigated by incorporating CholEsteryl BODIPY™ in the nanoparticles. Thus, SqCsA, SqCsA/**3a** 95:5 and 75:25 fluorescent NPs were prepared using the same method as described above, by adding 1% (*w*/*w*) of CholEsteryl BODIPY™ C11 to the ethanolic phase. 20,000 cells per well (MCEC) were seeded in 8-well Ibidi plates and allowed to adhere for 24 h. Cells were then incubated with 30 µg/mL of fluorescently labeled NPs for 2, 18, and 24 h in a 5% CO_2_ humidified incubator at 37 °C. After incubation, cells were washed with PBS, fixed with 4% paraformaldehyde (PFA) for 10 min and permeabilized with 0.1% Triton™ X-100 for 3 min. The actin cytoskeleton was stained with phalloidin for 2 h at room temperature, and the nuclei were stained with DAPI contained in the mounting medium (Tebu-bio) before observation. Images were acquired using an inverted Confocal Laser Scanning Microscope (CLSM) Leica TCS SP8 (Leica Microsystems, Wetzlar, Germany) with an HC PL APO CS2 63×/1.40 oil immersion objective lens. The instrument was equipped with a 405 nm diode for DAPI (nuclei) excitation and a WLL Laser set at 488 nm excitation for phalloidin-Atto 488 and 542 nm for CholEsteryl BODIPY™ NPs. Blue, green, and red fluorescence emissions were collected, respectively, with 410–460, 495–550, and 560–640 nm wide emission slits using a sequential mode and with multialkali PMT (blue and green channels) and Internal Hybrid Detector (HyD) for red channel with time gating set from 0.3 to 5.9 ns. The pinhole was set at 1.0 Airy unit, giving an optical slice thickness of 0.89 µm. The detection offset was chosen to a small number of zero-value pixels, and detector gains were set to optimize the dynamic range while ensuring minimal saturated pixels (based on the most fluorescent sample) and were kept for all acquisitions of the same experiment. Twelve-bit numerical images were acquired using Leica SP8 LAS X software (Version 3.5.5; Leica, Microsystems, Wetzlar, Germany). For nanoparticles fluorescence analysis, we specifically developed an image-processing macro with the FIJI software (Fiji Is Just, ImageJ 2.3.0/1.53t; Java 1.8.0_172) (https://imagej.net/Fiji), accessed on 14 September 2022 [23] using the «Freehand selection» tool to determine each cell outlines and «Thresholding» to limit measure to region of interest (nanoparticle’s fluorescence). Values were exported to Excel for further analysis and graphical representation.

### 2.10. Determination of Antioxidant Capacity

The antioxidant capability of SqCsA/**3a** NPs was determined using the Oxygen Radical Activity Capacity (ORAC) assay kit (ab 233473) according to the method described in the kit. Briefly, SqCsA/**3a** NPs at 2 different ratios (75:25 and 95:5) were diluted to obtain a final concentration of 12 or 60 µg/mL. Twenty-five µL of sample, phosphate buffer (blank), or Trolox standard were added in triplicate in a 96 well plate. Then, 150 µL of fluorescein solution was added to each well and incubated at 37 °C for 30 min. The peroxide radicals were produced by adding 25 µL of 2,2′-azobis(2-amidinopropane) dihydrochloride (AAPH) just before plate reading. Fluorescence was measured every 2 min for 1 hour using a microplate reader (ex/em: 485/535 nm). A calibration curve of Trolox in the concentration range 0 to 100 µM was used in each plate read. 

### 2.11. Statistical Analysis 

Data were reported as mean ± standard deviation (SD). Statistical differences between two groups were assessed using the unpaired Student t test with the GraphPad Prism 7.0 software (GraphPad Software, Inc., San Diego, CA, USA). A value of *p* ≤ 0.05 was deemed significant.

## 3. Results and Discussion

### 3.1. Synthesis of the Lipid-Cys-RYKF Bioconjugates

To embed the SS-31 peptide with self-assembling ability, a small library of polyisoprenyl conjugates were designed, adding a cysteine residue on its *N*-terminal extremity to anchor the lipid side chain through maleimide/thiol chemistry (Figure 1). It has been previously demonstrated that such modification of the SS-31 peptide did not impair the biological properties of the peptide, while strongly enabling the conjugation, thanks to the easy thiol-Michael reaction of maleimide with thiols [12]. Thus, at the outset of this study we decided to first explore the introduction of a simple C_32_-squalenoyl chain [21]. The SS-31 peptide being quite expensive due to the presence of non-canonical (D)-arginine and 2,6-dimethyl tyrosine amino-acids, the preliminary chemical and physico-chemical explorations were first conducted using a cheaper surrogate containing (L)-arginine and (L)-tyrosine (Cys-Arg-Tyr-Lys-PheNH_2_ (**2b**, Figure 1)). We first targeted the maleimide **8** in which the squalene chain was bound to the reactive polar head through a diethylene glycol spacer to move the reaction center of the peptide away from the highly lipophilic chain. This compound was previously obtained through ester bond formation from C_32_SqCO_2_H and 1-[2-(2-hydroxyethoxy)ethyl]-maleimide [24]. It was found that a more efficient route to this material involved the coupling of the C_32_SqCO_2_H with the known azatricyclo derivative **6** [22] (Figure 2) followed by unmasking of the sensitive maleimide double bond by retro-Diels-Alder reaction in refluxing toluene. Accordingly, the maleimide squalene derivative **8** was obtained in 34% overall yield. With the aim of modulating their amphiphilic balance, two other bioconjugates were synthetized. Solanesol (**13**) is a nonaprenol extracted from tobacco leaves. It was previously coupled to highly hydrophilic siRNA to provide stable nanoparticles [25]. With a regular polyisoprenoid chain of 45 carbons, solanesol was expected to increase the hydrophobic character of the conjugate with respect to the SqC_32_. Furthermore, the presence of the free hydroxyl group allowed a straightforward access to the *N*-solanesyl maleimide **14** through Mitsunobu reaction with maleimide [26]. A more lipophilic derivative was further prepared introducing two C_27_-squalene chains positioned on the two hydroxylethyl appendages of the diethanolamine moiety of **10**. Thus, reaction of 2,2′-[(2-aminoethyl)imino]diethanol (**9**) with the known 7-oxabicyclo [2.2.1]hept-5-ene-2,3-exo-dicarboxylic anhydride **5** [27] provided the diol **10** which was further engaged in a double esterification with C_27_SqCO_2_H to give the bis-ester **11**. Unmasking of the maleimide by refluxing in toluene as described previously afforded the bis-squalene maleimide **12** in 10.4% overall yield over the three steps.

With maleimides **8**, **12**, **14** in hand, the coupling with the model peptide Cys-Arg-Tyr-Lys-PheNH_2_ (**2b**) was addressed. The main challenge to achieve this task was to find a suitable solvent to dissolve both entities, since the peptide as a tris(trifluoroacetate) salt was only soluble in water while on the other hand, the highly lipophilic maleimides **8**, **12**, **14** were almost insoluble in most polar solvents, including water. We finally found that DMF was a suitable solvent to dissolve both compounds. In the event, stirring at room temperature of the peptide **2b** with a stoichiometric amount of maleimides **8**, **12**, **14** in DMF afforded the desired conjugates in quantitative yields after removal of the solvent under vacuum. The desired conjugates **3a, 4a**–**c** (Figure 2) were obtained in quantitative yield without need of further purification. Full characterization of the bioconjugates was achieved by ^1^H and ^13^C NMR, including 2D experiments COSY, HMQC, HMBC and NOESY. In all cases, the conjugates were obtained as a 1:1 mixture of two diastereomers at the C-3 maleimide carbon center due to the lack of stereoselectivity in the thiol-Michael addition. Despite the complexity of the spectra, the NMR revealed the presence of the vinyl and methyl protons of the polyisoprenyl chain together with the characteristic protons of the peptide backbone. Furthermore, a split ABX system for the 3-thio-succinimide system was observed in agreement with conjugate addition of the cysteine sulfur atom on the maleimide moiety. Mass spectrometry confirmed the NMR assignments. Significantly, mono-, bi- and tri-charged ions were detected in positive ESI mode for adducts **3a**–**c**, corresponding to the protonation of the three basic functions of Arg, Lys, and N-terminal Cys residues. Having secured a viable synthetic route to the polyisoprenoyl conjugates, we turned our attention to conjugation of the Cys-SS-31 (**2a**) peptide. With this material, we focused on the C_32_ squalenic acid which appeared to provide the optimal physico-chemical properties for the nanocarrrier (see Section 3.3.). According to the method used to get conjugate **4a**–**c** (Figure 1), the condensation of peptide **2a**, which displays the (D)-arginine and 2,6-dimethyl tyrosine found in elamipretide, with maleimide **8** in DMF afforded the conjugate **3a** (Figure 1) in quantitative yield. The latter was fully characterized by ^1^H, ^13^C NMR and mass spectrometry as for the models conjugates **4a**–**c**.

### 3.2. Nanoparticle Obtention

**Figure 3:**
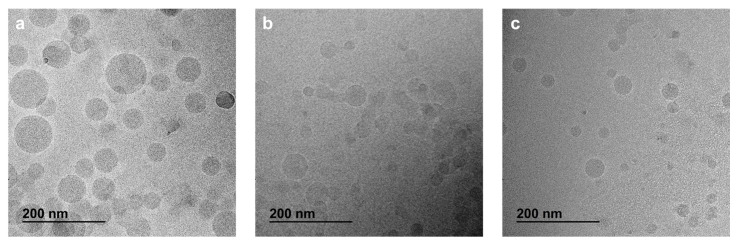
CryoTEM images of SqCsA/**4a** (**a**), **4b** (**b**) and **4c** (**c**) NPs.

**Figure 4:**
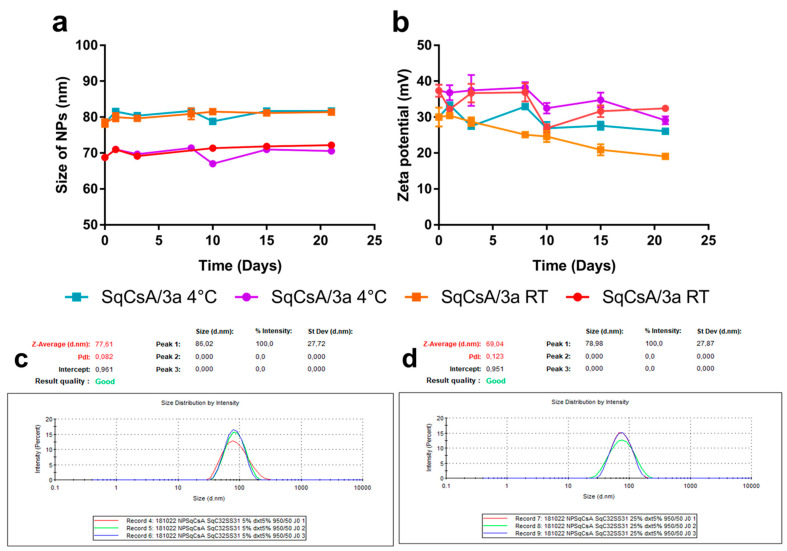
Colloidal stability of SqCsA/**3a** NPs in size (**a**) and zeta potential (**b**) comparing ratio and storage conditions (4 °C and RT). Circle and squares correspond, respectively, to ratio 75:25 and 95:5. Particle size distribution of SqCsA/3a NPs at 95:5 (**c**) and 75:25 (**d**) ratios.

In our hand, pure **4a**, **4b** and **4c** bioconjugates did not result in nanoparticle formation upon nanoprecipitation. However, when co-nanoprecipitated with SqCsA at therapeutic ratios, the bioconjugates formed stable nanoparticles (NPs) at different ratios, based on the concentration used in clinical trials [10,28,29] (Table 1). The obtained NPs at different ratios were found stable for at least 2 days under various storage conditions (Figure S1), except for the 98:2 ratio that aggregated rapidly after solvent evaporation. The NPs had a size between 56 and 88 nm, a positive zeta potential between 34 and 54 mV, and a good polydispersity index except for SqCsA/**4c** (Table 1). The addition of **4a**, **4b**, and **4c** bioconjugates resulted in a decrease in size compared to SqCsA and squalenic acid NPs as well as a switch in zeta potential [20,30]. The change from negative to positive zeta potential was expected due to the positive charges of the peptide **2b,** and was previously observed for other squalenoylated NPs [31,32] and for NPs containing elamipretide [12]. Furthermore, it appeared that the NPs tended to be larger when the hydrophobic part of the peptide bioconjugate was smaller and when the ratio of peptide bioconjugate was lower. The drug loading of peptide **2a** or **2b** within the multidrug NPs was calculated based on the ratio of bioconjugate **4a**–**c** and **3a** in the NPs and the molar ratio of peptide **2a** or **2b** compared to the molar mass of the bioconjugate **4a**–**c** and **3a**. We were therefore able to develop hybrid NPs using biocompatible lipids with suitable sizes and PDI, as well as a higher drug loading than previously reported [14]. These NPs were further characterised using cryogenic transmission electron microscopy (CryoTEM), revealing a monodisperse population of spherical NPs (Figure 3). Finally, we obtained NPs co-encapsulating SqCsA and **3a** bioconjugate as previously described. These NPs were smaller in size than those encapsulating SqCsA and bioconjugate **4a,** and were stable for at least 21 days regarding the ratio and storage conditions (Figure 4).

### 3.3. Cytotoxicity of SqCsA/***4a***, ***4b*** and ***4c*** NPs

**Figure 5:**
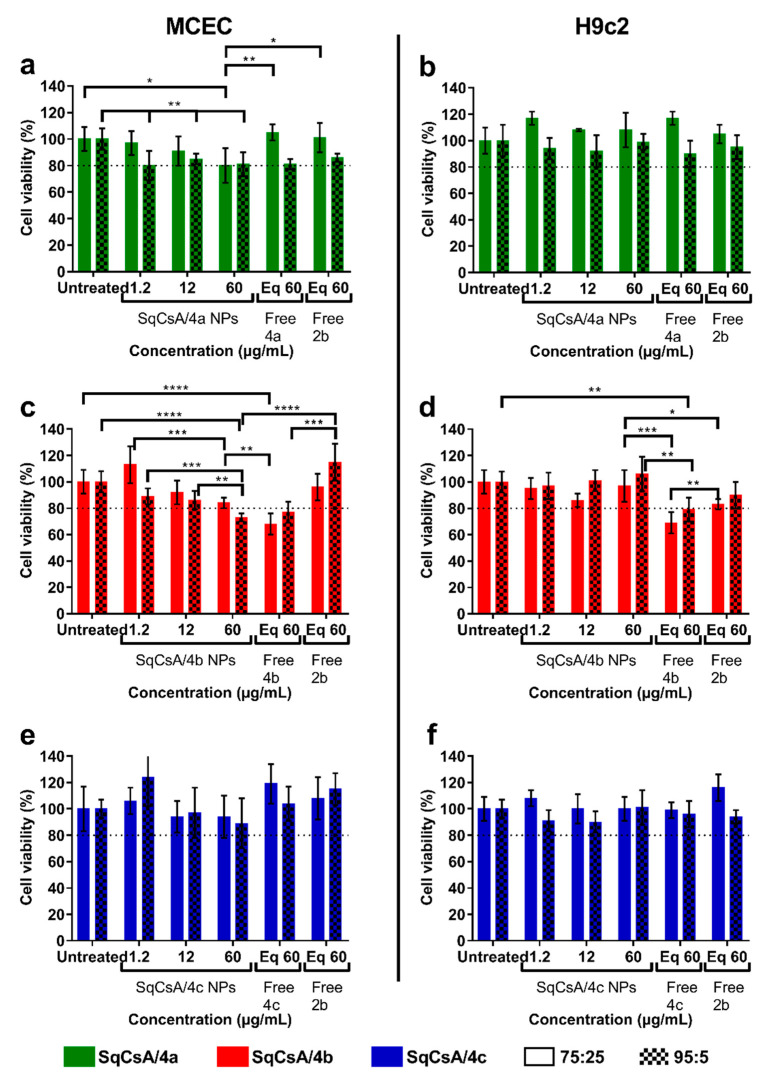
Cell viability assessment of Mouse Cardiac Endothelial Cells (MCEC) (**a**,**c**,**e**) and H9c2 (**b**,**d**,**f**) cells treated either with SqCsA/**4a** (**a**,**b**), **4b** (**c**,**d**) or **4c** (**e**,**f**) NPs. Cell viability was expressed as a percentage of the viability of untreated cells. An equivalent concentration (eq) of free bioconjugate **4a** (**a**,**b**), **4b** (**c**,**d**) or **4c** (**e**,**f**) or the free peptide (**2b**) contained in the nanoparticles at the highest concentration was used as control. * *p* ≤ 0.05, ** *p* ≤ 0.01, *** *p* ≤ 0.001, **** *p* ≤ 0.0001.

To determine the cytotoxicity of SqCsA/**4a**, **4b** and **4c** NPs on MCEC and H9c2 cell lines, an MTT assay was performed using different concentrations of NPs at 2 ratios. A dose equivalent to the highest dose of the peptide bioconjugates and the free peptide were used as controls. Except for SqCsA/**4b** NPs, cell viability was high above 80%, for all cell lines and ratios (Figure 5), demonstrating that these NPs have low cytotoxicity at the doses used. The peptide **2b** showed no significant cytotoxicity compared to untreated cells in the MCEC and H9c2 cell lines, which is consistent with previous results obtained at a higher dose for this peptide on H9c2 cells [33], endothelial cells [14], or other cells [34].

**Figure 6:**
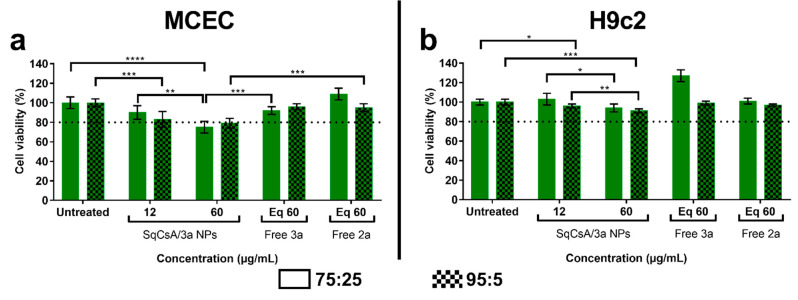
Cell viability assessment of Mouse Cardiac Endothelial Cells (MCEC) (**a**) and H9c2 (**b**) cells treated with SqCsA/**3a** nanoparticles. Cell viability was expressed as a percentage of the viability of untreated cells. An equivalent concentration (eq) of free bioconjugate **3a** and the free peptide (**2a**) contained in the nanoparticles at the highest concentration was used as control. * *p* ≤ 0.05, ** *p* ≤ 0.01, *** *p* ≤ 0.001, **** *p* ≤ 0.0001.

C_32_ squalene acid was found to be the best candidate for further experiments based on polydispersity index (below 0.2) and cell viability (above 80%). Furthermore, the cytotoxicity of SqCsA/**3a** NPs was also studied on both cell lines and the results were similar to those obtained for SqCsA/**4a** NPs with a slight cytotoxicity at higher doses (Figure 6). Furthermore, cytotoxicity appeared to be significantly higher for an equivalent of SqCsA NPs or free CsA compared to SqCsA/**3a** NPs on both cell lines (Figure S2). Overall, cell viability appeared to be lower with the 95:5 ratio than with the 75:25 ratio, indicating that NP cytotoxicity may be the result of SqCsA NPs toxicity, which has been demonstrated previously by our group [20].

### 3.4. Surface Composition of NPs

**Figure 7:**
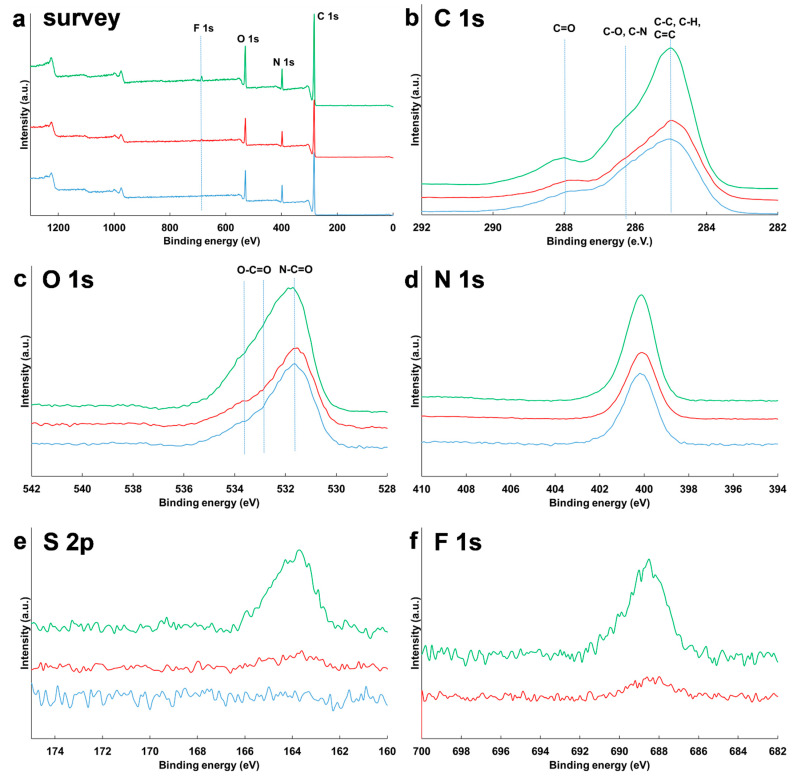
High resolution XPS spectra of SqCsA (blue), SqCsA/**3a** 95:5 (red), and SqCsA/**3a** 75:25 (green) are represented for survey spectra (**a**), Carbon C 1s (**b**), Oxygen O 1s (**c**), Nitrogen N 1s (**d**), Sulfur S 2p (**e**), and Fluorine F 1s (**f**).

High resolution spectra of C 1s, O 1s, N 1s, S 2p, and F 1s of NPs of SqCsA, SqCsA/**3a** 95:5 and 75:25 are shown in Figure 7, displaying the XPS survey spectra obtained on the three analyzed samples. C, N and O are evidenced in all three spectra, and F and S were detected in the SqCsA/**3a** 95:5 and 75:25 samples, suggesting the presence of the bioconjugate **3a** on the surface of the NPs. C 1s core level presented three components at 285 eV: C-C, C-H bonds, 286.4 eV: C-O, C-N bonds and 288 eV: C=O bonds. O 1s core level also presented three components at 531.5 eV: N-C=O bonds, 532.7eV and 533.8 eV: O=C-O bonds. The N 1s peak at 400.2 eV was associated to N in molecules. S 2p and F 1s peaks at 163.9 eV and 688.6 eV, respectively, were associated to S and F in the bioconjugate **3a**. The C 1s and O 1s envelopes showed substantial changes after co-nanoprecipitation with **3a** and S and F were detected on the surface. The intensity of the S 2p and F 1s peaks was higher for the SqCsA/**3a** 75:25 samples compared to the 95:5, suggesting that the bioconjugate **3a** quantity at the surface of the NP is higher. Atomic composition of molecules deduced from XPS peaks area were compared to theoretical values (Table S1). For the SqCsA, experimental and theoretical values obtained with Thermo Electron software were in good agreement. After co-nanoprecipitation with bioconjugate **3a**, the atomic ratio F/S was, respectively, 9 and 8.5 for SqCsA/**3a** 95:5 and 75:25 in good agreement with the theoretical value, 9. For SqCsA/**3a** 75:25, the theoretical atomic composition of the nanoparticle surface was different from the experimental composition, which may indicate an accumulation of the bioconjugate **3a** on the surface of the NPs. Knowing that the theoretical N/F atomic value was 11/9 in the bioconjugate **3a**, atomic percentages of SqCsA/**3a** have been calculated from the N 1s peak area. 5.5%at. of the bioconjugate **3a** and 94.5%at. of SqCsA were present on the SqCsA/**3a** 95:5 surface and 28.4%at. of the bioconjugate **3a** and 71.6%at. of SqCsA in the SqCsA/**3a** 75:25.

### 3.5. Cell Uptake of Multidrug Nanoparticles

**Figure 8:**
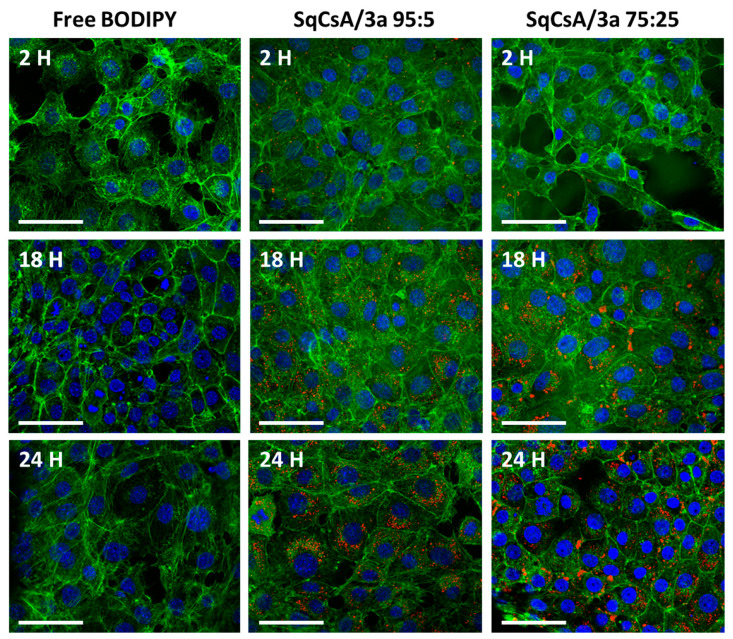
Uptake assessment of multidrug NPs by MCEC cells for two ratios (95:5 and 75:25). Cells incubated with only CholEsteryl BODIPY™, at an equivalent concentration to 30 µg/mL of NPs, were used as control and imaged at 2,18 and 24 h. Nucleus, actin filaments and NPs are represented, respectively, in blue, green, and red. Scale bar = 50 µm.

To understand whether the bioconjugate **3a** change the biological fate of multidrug NPs, the uptake of the SqCsA/**3a** multidrug NP was evaluated using MCEC cell line. MCEC cells were incubated at 2, 18, and 24 h with SqCsA/**3a** NP 95:5 or 75:25 tagged with CholEsteryl BODIPY™ C11 and analyzed using CLSM (Confocal Laser Scanning Microscopy). After 2 h of incubation, confocal images revealed no cell fluorescent signal in the free BODIPY while there was weak signal in the cells treated with the multidrug NPs. Over time, fluorescent signal increased indicating an internalization and accumulation of the NP inside the cells (Figure 8).

**Figure 9:**
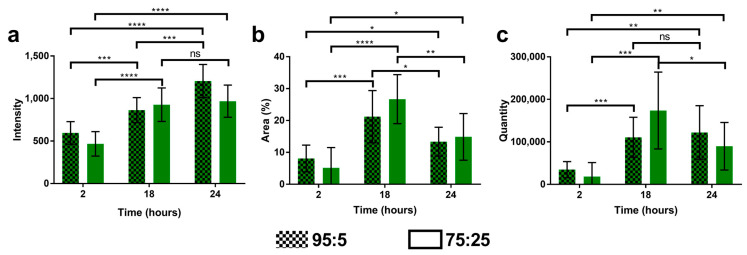
Quantification of fluorescence signal at 2,18 and 24 h for (**a**) intensity, (**b**) surface area, (**c**) quantity. MCEC cells were incubated with SqCsA/3a NPs at two ratios (95:5 and 75:25). Non-Significant (ns) *p* > 0.05, * *p* ≤ 0.05, ** *p* ≤ 0.01, *** *p* ≤ 0.001, **** *p* ≤ 0.0001.

The intensity and surface area of the fluorescence signal of the NPs were quantified, allowing the quantity of NPs inside the cells to be calculated (surface area*intensity) (Figure 9). According to these data, the intensity signal of the SqCsA/**3a** NPs increased over time, except between 18 and 24 h for the highest ratio of bioconjugate **3a** 75:25. The surface area of the NPs increased up to 18 h then decreased, indicating either aggregation, excretion, or degradation of the NPs. Concerning the quantity of NPs, there was an increase up to 18 h then a decrease, except for the SqCsA/**3a** NPs at a ratio of 95:5. This may indicate that the nanoparticles are not degraded and accumulate inside the cells.

### 3.6. Antioxidant Capacity

The antioxidant capacity of the SqCsA/**3a** NPs was assessed using an ORAC test. This method involves the determination of the area under the curve (AUC) calculated by integrating the decrease in fluorescence, to be compared with the Trolox calibration curve (Figure S3) to obtain an antioxidant capacity equivalent to a Trolox concentration (Table 2). The results presented in Table 2 showed a higher antioxidant capacity for SqCsA/**3a** NP 75:25 containing the higher amount of Cys-SS-31 peptide within the multidrug NP which is responsible for the antioxidant capacity.

It may be noticed that there was almost no difference in antioxidant capacity for the multidrug NPs at the 75:25 ratio and the free bioconjugate **3a**. Because encapsulation usually significantly reduces the antioxidant activity as observed, for example, for solid-liquid nanoparticle encapsulating curcumin [35,36], the results reported in Table 2 confirmed the surface coating of the NPs with the peptide as suggested by XPS analysis. This could be due to the localisation of the bioconjugate **3a** on the surface of the NP, which means that the peptide may still be accessible to exert its antioxidant activity. Moreover, the difference in antioxidant capacity between the free peptide **2a** and bioconjugate **3a** is probably due to the activity of the thiol group of the cysteine linker in peptide **2a.**

## 4. Conclusions

To conclude, in this study we synthesized three bioconjugates of elamipretide using three polyisoprenyl lipid chains with increasing hydrophobicity. Co-nanoprecipitation of the different bioconjugates in combination with SqCsA bioconjugate afforded stable nanoparticles without the need of any surfactant. It was found that the squalene acetic appendage (**3a**, **4a**) provided the optimal combination in terms of size, stability, and cytotoxicity of the nanoparticles. Surface analysis with XPS revealed that a significant amount of the elamipretide coated the surface of the NPs in line with their positive surface charges while keeping the antioxidant activity of the NPs. The simultaneous administration of both drugs within the same NP could be interesting as a new approach to reduce I/R injuries.

## Figures and Tables

**Table 1:** Size, Polydispersity index (PDI), Zeta potential and Drug loading of **2a** or **2b** for SqCsA/**4a**, **4b**, **4c** or **3a** NPs at different ratios.

	Ratios	Size (nm)	PDI	Zeta (mV)	Drug Loading (%)
SqCsA/**4a**	95:5	86 ± 31	0.129	+43 ± 15	3.1
	90:10	88 ± 31	0.124	+37 ± 12	6.2
	75:25	79 ± 30	0.141	+47 ± 16	15.6
SqCsA/**4b**	95:5	68 ± 27	0.161	+36 ± 11	3.0
	90:10	68 ± 27	0.160	+44 ± 12	6.0
	75:25	62 ± 27	0.183	+36 ± 13	14.7
SqCsA/**4c**	95:5	66 ± 29	0.195	+35 ± 14	2.6
	90:10	64 ± 29	0.208	+54 ± 8	5.2
	75:25	56 ± 26	0.217	+34 ± 38	12.9
SqCsA/**3a**	95:5	78 ± 26	0.111	+30 ± 8	3.2
	75:25	69 ± 25	0.134	+37 ± 5	15.8

**Table 2:** Trolox equivalent (TE) of SqCsA/**3a** NPs, **3a** bioconjugate and **2a** peptide.

Type of Molecule		Ratio	
		95:5	75:25
SqCsA/**3a** NPs	12 µg/mL	0.107	10.392
	60 µg/mL	2.144	49.796
Free **3a** (bioconjugate)		10.715	47.557
Free **2a** (peptide)		14.065	66.667

## Data Availability

Not applicable.

## Supplementary Materials

The following supporting information can be downloaded at: https://www.mdpi.com/article/10.3390/ma16051812/s1, Figure S1. Colloidal stability comparing ratio (a) and storage conditions (b). a: Circle and squares correspond, respectively, to ratio 75:25 and 95:5. b: Triangles and stars correspond, respectively, to storage of 4 °C and room temperature (RT); Table S1. Experimental (exp) and Theoretical (th) atomic composition of SqCsA, SqCsA/**3a** 95:5 and 75:25. The atomic composition is represented by a percentage of carbon (C), oxygen (O), nitrogen (N), sulfur (S) and fluorine (F); Figure S2. Cell viability of SqCsA/**3a**, SqCsA NPs and free CsA on MCEC and H9c2 cell lines; Figure S3. Trolox calibration curve and equation.
