# Supplementary material for: Development and Characterization of Innovative Multidrug Nanoformulation for Cardiac Therapy
Source: Materials (Basel). 2023 Feb 22;16(5):1812. doi: 10.3390/ma16051812 (PMC10003764; doi:10.3390/ma16051812)
Supplement: Supplementary file 1 [file materials-16-01812-s001.zip › materials-2158663-supplementary.pdf]

materials

MDPI

Supplementary Materials

Development and Characterization of Innovative Multidrug Nanoformulation for Cardiac Therapy

Amandine Gendron [1], Séverine Domenichini [2], Sandrine Zanna [3], Frédéric Gobeaux [4], Christophe Piesse [5], Didier Desmaële [1,*] and Mariana Varna [1,*]

Amandine Gendron [1], Séverine Domenichini [2], Sandrine Zanna [3], Frédéric Gobeaux [4], Christophe Piesse [5], Didier Desmaële [1,*] and Mariana Varna [1,*]

[1] Institut Galien Paris-Saclay, CNRS UMR 8612, Université Paris-Saclay, 91400 Orsay, France; amandine.gendron@universite-paris-saclay.fr

[2] UMS-IPSIT Plateforme MIPSIT, Université Paris-Saclay, CNRS, Inserm, Ingénierie et Plateformes au Service de l'Innovation Thérapeutique, 91400 Orsay, France; severine.domenichini@universite-paris-saclay.fr

[3] Research Group Physical Chemistry of Surfaces, Chimie ParisTech-CNRS, Institut de Recherche de Chimie Paris, PSL Research University, 11 Rue Pierre et Marie Curie, 75005 Paris, France; sandrine.zanna@chimieparistech.psl.eu

[4] CEA, CNRS, NIMBE, Université Paris-Saclay, 91191, Gif-sur-Yvette, France; frederic.gobeaux@cea.fr

[5] CNRS, Institut de Biologie Paris-Seine (IBPS), Plateforme d'Ingénierie des Protéines—Service de Synthèse Peptidique, Sorbonne Université, 75006 Paris, France; christophe.piesse@sorbonne-universite.fr

* Correspondence: didier.desmaele@universite-paris-saclay.fr (D.D.); mariana.varna-pannerec@universite-paris-saclay.fr (M.V.)

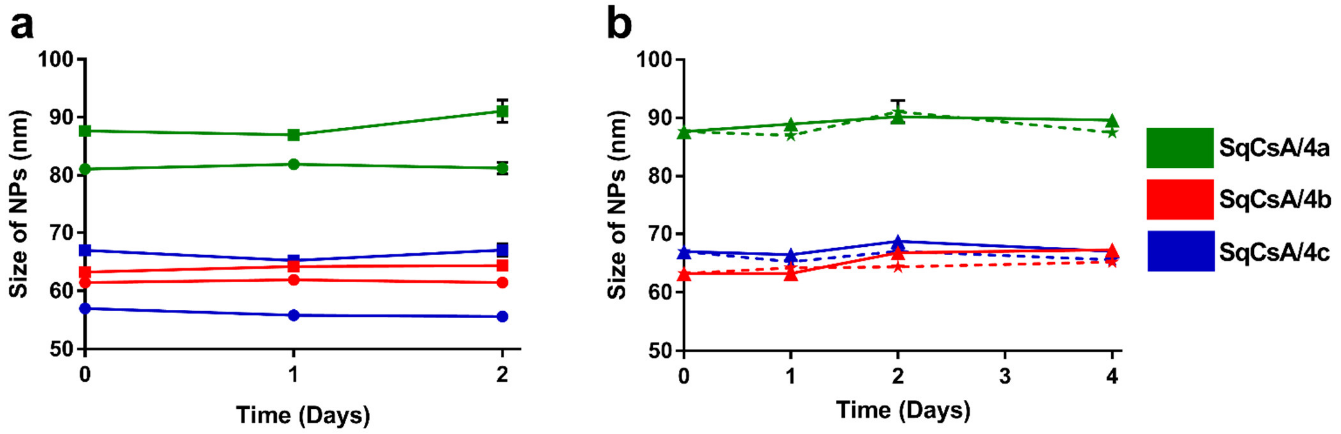

Figure S1. Colloidal stability comparing ratio (**a**) and storage conditions (**b**). a: Circle and squares correspond respectively to ratio 75:25 and 95:5. b: Triangles and stars correspond respectively to storage of 4 °C and room temperature (RT).

Table S1. Experimental (exp) and Theoretical (th) atomic composition of SqCsA, SqCsA/**3a** 95:5 and 75:25. The atomic composition is represented by a percentage of carbon (C), oxygen (O), nitrogen (N), sulfur (S) and fluorine (F).

%at.	C	O	N	S	F	F/C
**SqCsA (exp)**	76.6	12.6	10.8			
**SqCsA (th)**	77.8	12.8	9.4			
**SqCsA/3a 95:5 (exp)**	76.2	13.3	10	0.05	0.45	9
**SqCsA/3a 95:5 (th)**	77.2	12.9	9.4	0.04	0.38	9
**SqCsA/3a 75:25 (exp)**	71.4	16.6	9.5	0.26	2.2	8.5
**SqCsA/3a 75:25 (th)**	75.2	13.2	9.4	0.21	1.9	9

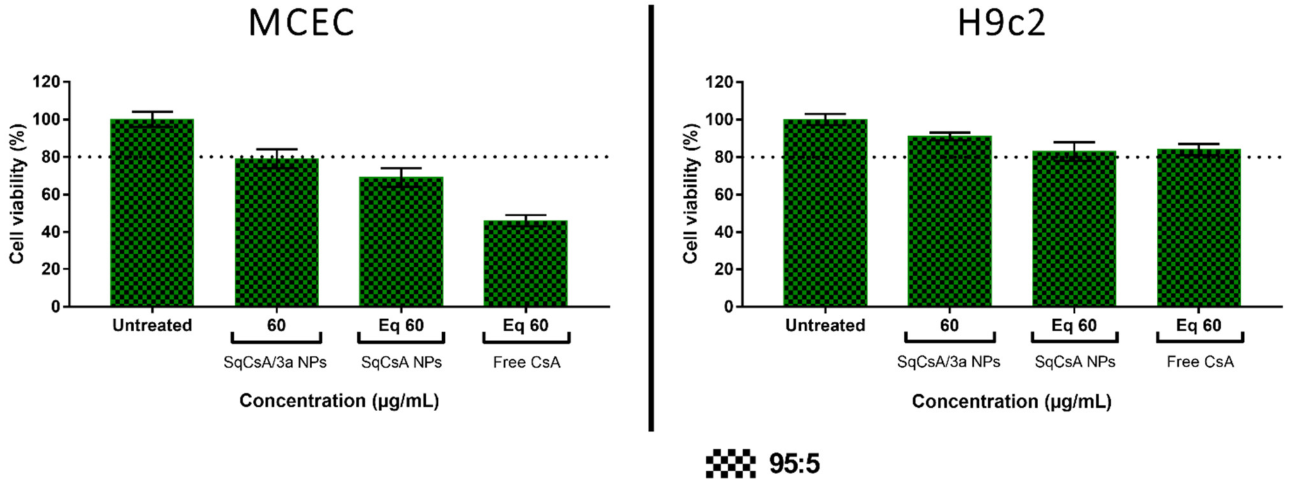

Figure S2. Cell viability of SqCsA/3a, SqCsA NPs and free CsA on MCEC and H9c2 cell lines.

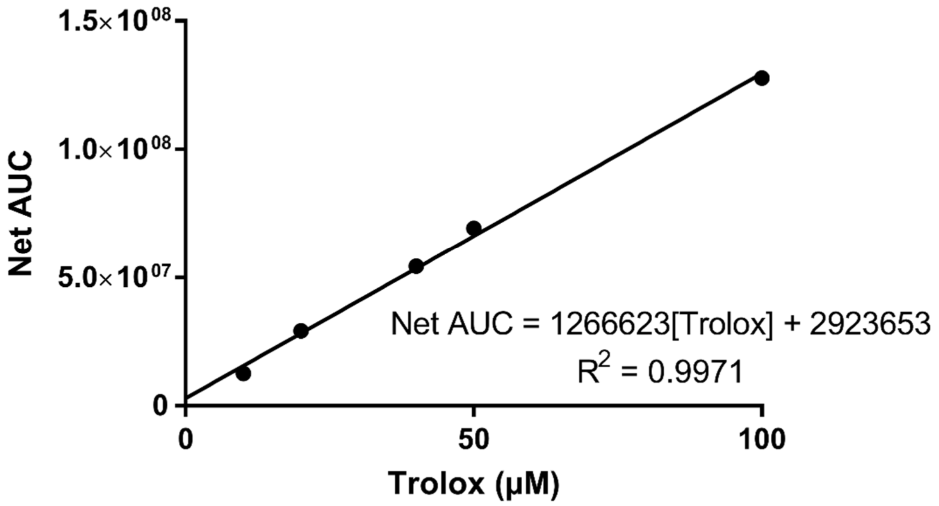

Figure S3. Trolox calibration curve and equation.
